# MiRNA160 is associated with local defense and systemic acquired resistance against *Phytophthora infestans* infection in potato

**DOI:** 10.1093/jxb/ery025

**Published:** 2018-01-30

**Authors:** Bhavani Natarajan, Harpreet S Kalsi, Prajakta Godbole, Nilam Malankar, Aarthy Thiagarayaselvam, Sundaresha Siddappa, Hirekodathakallu V Thulasiram, Swarup K Chakrabarti, Anjan K Banerjee

**Affiliations:** 1 Biology Division, Indian Institute of Science Education and Research (IISER Pune), Pune, Maharashtra, India; 2 National Chemical Laboratory (NCL) Pune, Maharashtra, India; 3 Central Potato Research Institute (CPRI),Shimla, Himachal Pradesh, India

**Keywords:** Auxin–salicylic acid antagonism, microRNA, miR160, *Phytophthora infestans*, *Solanum chacoense*, *Solanum tuberosum*, systemic acquired resistance

## Abstract

To combat pathogen infection, plants employ local defenses in infected sites and elicit systemic acquired resistance (SAR) in distant tissues. MicroRNAs have been shown to play a significant role in local defense, but their association with SAR is unknown. In addition, no such studies of the interaction between potato and *Phytophthora infestans* have been reported. We investigated the role of miR160 in local and SAR responses to *P. infestans* infection in potato. Expression analysis revealed induced levels of miR160 in both local and systemic leaves of infected wild-type plants. miR160 overexpression and knockdown plants exhibited increased susceptibility to infection, suggesting that miR160 levels equivalent to those of wild-type plants may be necessary for mounting local defense responses. Additionally, miR160 knockdown lines failed to elicit SAR, and grafting assays indicated that miR160 is required in both local and systemic leaves to trigger SAR. Consistently, SAR-associated signals and genes were dysregulated in miR160 knockdown lines. Furthermore, analysis of the expression of defense and auxin pathway genes and direct regulation of *StGH3.6*, a mediator of salicylic acid–auxin cross-talk, by the miR160 target *St*ARF10 revealed the involvement of miR160 in antagonistic cross-talk between salicylic acid-mediated defense and auxin-mediated growth pathways. Overall, our study demonstrates that miR160 plays a crucial role in local defense and SAR responses during the interaction between potato and *P. infestans*.

## Teaser

Potato miR160 is crucial for both local and SAR responses to the late blight pathogen *Phytophthora infestans* and modulates antagonistic cross-talk between auxin-mediated growth and salicylic acid-mediated defense responses.

## Introduction

Upon infection with a pathogen, plants mount defense responses in both local infected and systemic non-infected sites. At the local site, plants recognize pathogen-associated molecular patterns (PAMPs) through their pattern recognition receptors and activate PAMP-triggered immunity (PTI) (Chisholm *et al.*, 2006). Pathogens release effector proteins into the plant cell to suppress PTI. In this ‘arms race’, plants have in turn evolved resistance proteins that can distinguish effectors and activate effector-triggered immunity (ETI) (Chisholm *et al.*, 2006; Dodds and Rathjen, 2010). The activation of local defense responses also induces systemic signals that result in broad-spectrum resistance at distant sites, termed systemic acquired resistance (SAR) (Kachroo and Robin, 2013). Salicylic acid (SA) is one of the well-studied and necessary components of the SAR process (Gaffney *et al.*, 1993; Wildermuth *et al.*, 2001). In Arabidopsis and tobacco, SA levels increase both locally and systemically upon SAR induction (Yalpani *et al.*, 1991; Enyedi *et al.*, 1992; Summermatter *et al.*, 1995). Furthermore, recent studies have identified methyl salicylate (MeSA) (Park *et al.*, 2007), azelaic acid (Jung *et al.*, 2009), glycerol-3-phosphate (Chanda *et al.*, 2011), dehydroabietinal (Chaturvedi *et al.*, 2012), and pipecolic acid (Návarová *et al.*, 2012) as potential mobile SAR signals. A lipid transfer protein, DIR1 (DEFECTIVE IN INDUCED RESISTANCE 1), was shown to be crucial for the induction of SAR by many of these mobile signals (Jung *et al.*, 2009; Liu *et al.*, 2011; Chanda *et al.*, 2011; Chaturvedi *et al.*, 2012).

In the past decade, microRNAs (miRNAs) have emerged as major contributors to the immune responses of plants, especially in PTI and ETI. miRNAs are small (~21–24 nt), endogenous, non-coding RNAs that act as negative regulators of gene expression (Jones-Rhoades *et al.*, 2006). In Arabidopsis, miR393 was shown to be involved in PTI responses against bacterial pathogens by facilitating the suppression of the auxin signaling pathway (Navarro *et al.*, 2006). Similarly, Arabidopsis miR160, miR398, and miR773 were shown to be associated with PTI (Li *et al.*, 2010), and tomato miR482 and miR5300 (Shivaprasad *et al.*, 2012; Ouyang *et al.*, 2014) and tobacco miR6019 (Li *et al.*, 2012) were demonstrated to play important roles in ETI. These reports have established the role of miRNAs in PTI and ETI responses of various plants. However, the role of miRNAs in the regulation of SAR is unknown.

SAR has been widely studied in the model plants Arabidopsis and tobacco; however, only a few studies have explored SAR in crop plants. Potato, the fourth most important food crop worldwide, is severely affected by late blight disease caused by the oomycete pathogen *Phytophthora infestans*, which leads to massive crop loss. Although much understanding has been gained regarding local defense responses and various resistance genes in potato (van Ooijen *et al.*, 2007), our knowledge of SAR in potato is still rudimentary. Currently, we understand only that SA is important for SAR induction (Yu *et al.*, 1997) and that MeSA acts as a mobile SAR signal in potato (Manosalva *et al.*, 2010). Furthermore, the role of miRNAs in the interaction between potato and *P. infestans* has not been investigated. In light of recent findings that effector proteins of *Phytophthora* spp. can suppress host small RNA-mediated defense responses (Qiao *et al.*, 2015; Ye and Ma, 2016), studying the role of potato miRNAs in mediating the local defense and SAR responses against *P. infestans* could be of great importance toward understanding late blight disease.

In this study, from an initial screening of 10 candidate miRNAs, we focused on the function of miR160. We show that the expression of miR160 is induced in both local and systemic leaves of wild-type (WT) potato upon *P. infestans* infection. Both overexpression (OE) and knockdown (KD) of miR160 result in enhanced susceptibility. Additionally, SAR and grafting assays on miR160 transgenic lines provide crucial insights regarding the role of miR160 in mounting an effective SAR response. Expression analysis of auxin and SAR genes and the interaction of *St*ARF10 protein with the *StGH3.6* promoter suggest the involvement of miR160 in auxin–SA antagonistic cross-talk. Overall, our findings indicate that miR160 plays an important role in modulating local defense and SAR responses in potato during *P. infestans* infection.

## Materials and methods

### Plant and pathogen growth conditions

All WT and transgenic potato (*Solanum chacoense* and *Solanum tuberosum* cv. Désirée) plants were grown and maintained as described previously (Banerjee *et al.*, 2006*b*). The oomycete pathogen *Phytophthora infestans* strain A2 was maintained at 18 °C in pea agar media, corn media, and potato slices, and confirmed by amplifying part of the internal transcribed spacer 2 (ITS2) ribosomal DNA using primer sets PINF and ITS5 (Trout *et al.*, 1997) (Supplementary Fig. S1A at *JXB* online). The bacterial pathogen *Ralstonia solanacearum* was maintained in nutrient agar medium and confirmed by performing PCR as described previously (Lee and Wang, 2000) (Supplementary Fig. S1B).

### *P. infestans* infection and arachidonic acid treatment

For all infection experiments, *P. infestans* sporangia were used at a concentration of 2 × 10^5^ ml^–1^ and treated plants were incubated at 18 °C and 90% humidity. Time-course expression analysis of miR160 and *StARF10* in *S. chacoense* was performed by inoculating 10 µl of sporangia solution on the abaxial side of the eighth to 11th leaves, counted from the top of the plant. Control plants were inoculated with sterile water. Inoculated local leaves (leaves 8–11) and non-inoculated systemic leaves (leaves 5–7) were harvested at 12, 24, 48, and 96 hours post-inoculation (hpi). For local infection analysis, *S. tuberosum* WT and miR160 transgenic plants were sprayed with *P. infestans* sporangia and disease progression was monitored for 14 days. Samples were collected on 0, 2, 5, 7, 9, 11, and 14 days post-inoculation (dpi) for various analyses. For arachidonic acid (AA) treatment analysis, plants were treated with 30 µl of 0.05 mM AA. Local (AA-treated) and systemic (AA-untreated) leaves were collected at 0, 24, 48, 72, and 96 hours post-treatment (hpt).

### Detection and qRT–PCR of miRNAs

A 1 µg quantity of total RNA was used for cDNA preparation using the respective stem-loop primers of miRNAs, followed by endpoint PCR or quantitative real-time (qRT)–PCR as described by Varkonyi-Gasic *et al.* (2007). *GAPDH* was used as normalization gene.

### Northern blot analysis

Northern blot analysis for miR160 and U6 was carried out following a previously described protocol (Rio, 2014) with minor modifications, such as capillary transfer of RNA to nylon membrane and a hybridization temperature of 30 °C. Probe details are provided in Supplementary Table S2.

### Prediction of miR160 targets and cleavage site mapping

To predict target genes of miR160 in potato, the target prediction software packages psRNATarget (http://plantgrn.noble.org/psRNATarget/; accessed 12/02/2018) (Dai and Zhao, 2011) and TAPIR (http://bioinformatics.psb.ugent.be/webtools/tapir/; accessed 12/02/2018) (Bonnet *et al.*, 2010) were used. The *S. tuberosum* transcript library (http://solanaceae.plantbiology.msu.edu/pgsc_download.shtml; accessed 12/02/2018) was used as the target database. For *in planta* validation of the miR160 targets *StARF10* and *StARF16,* a modified 5ʹ-RNA ligase-mediated rapid amplification of cDNA ends (RLM-RACE) technique was performed as described previously (Bhogale *et al.*, 2014).

### Gene expression analysis

For expression analysis of mRNA transcripts, 1 µg of total RNA was used for oligo(dT) cDNA preparation with Superscript-III Reverse Transcriptase (Invitrogen), and qRT–PCR reactions were carried out using KAPA SYBR Green Mix (Kapa Biosystems). Data were analyzed using the 2^−∆Ct^ method (Livak and Schmittgen, 2001) for all the experiments except the AA treatment experiment, in which the 2^−∆∆Ct^ method was used. *GAPDH* was used as the normalization gene in all experiments.

### miR160 overexpression and knockdown construct generation

For the generation of an miR160 OE construct, pBI121-35S::pre160 cDNA was prepared from potato leaf RNA using oligo(dT) primers. The miR160 precursor (*St-pre160*) was amplified and cloned into the binary vector pBI121 under the 35S CaMV constitutive promoter. For KD lines, the endogenous target mimicry (eTM) approach was employed using the construct pCAMBIA-35S::ath-eTM160 (Wu *et al.*, 2013). Plant transformation was performed as described previously (Banerjee *et al.*, 2006*b*).

### *P. infestans* DNA quantification

For absolute quantification, a standard curve for the *P.infestans*-specific sequence O8 was generated using different concentrations of *P. infestans* genomic DNA (Supplementary Fig. S12). Genomic DNA was isolated from leaf samples collected from *P. infestans*-infected plants at 14 dpi. 50 pg of DNA was used for qRT–PCR analysis of the O8 sequence using the primers O8-3 and O8-4 (Judelson and Tooley, 2000).

### SAR and grafting assays

Four-week-old plants were subjected to primary infection with *P. infestans* followed by secondary infection with *R. solanacearum*. For the primary infection, 50 µl of 2 × 10^5^ ml^−1^*P. infestans* sporangia was swabbed on to the two lowest leaves and plants were incubated at 18 °C. Mock inoculation was carried out with sterile water. Four days after primary infection, three upper leaves were infiltrated with 10^6^ colony-forming units ml^−1^ (OD_600_ ~0.1) of *R. solanacearum* and plants were incubated at 28 °C. After 5 days of secondary infection, a 1 cm^2^ piece of leaf tissue was excised and crushed in sterile water. The sample was serially diluted and plated on nutrient agar medium, and the bacterial count was recorded. For grafting analysis, two types of homografts (WT/WT and eTM160-26/eTM160-26) and heterografts (WT/eTM160-26 and eTM160-26/WT) were generated following the protocol described by Banerjee *et al.* (2006a). SAR assays were performed as described above. In brief, two leaves of grafted stock plants were inoculated with either *P. infestans* or sterile water (mock treatment). Four days after the primary inoculations, systemic scion leaves of all the grafts were infiltrated with *R. solanacearum*. After 5 days of secondary infection, systemic scion leaves were harvested and the bacterial count was recorded.

### Quantification of salicylic acid

For quantification of SA levels, modification of a previous protocol was followed (Forcat *et al.*, 2008). A sample of 50 mg of ground leaf tissue was extracted in 400 µl of 10% methanol containing 1% glacial acetic acid. The mixture was vigorously vortexed and incubated on ice for 30 min, followed by centrifugation at 13000 *g* for 10 min at 4 °C to obtain the supernatant. This process was repeated once and the supernatant volume was adjusted to 1 ml using a volumetric flask. Samples were resolved through a Thermo Scientific Hypersil Gold column of particle size 1.9 μm and dimensions 50 × 2.1 mm with a flow rate of 0.2 ml min^–1^ and a gradient solvent program of 10 min (0.0 min, 10% methanol/water; 0.5 min, 10% methanol/water; 3.0 min, 50% methanol/water; 10 min, 50% methanol/water). Formic acid (0.1% LC-MS grade) was also added to the water. MS and MS/MS experiments were performed in electrospray ionization (ESI)-negative ion mode using the tune method: sheath gas flow rate 45, auxiliary gas flow rate 10, sweep gas flow rate 2, spray voltage (|KV|) 3.60, spray current (μA) 3.70, capillary temperature 320 °C, s-lens RF level 50, heater temperature 350 °C. ESI-MS data were recorded in full scan mode within the mass range *m/z* 100–1000 (Supplementary Fig. S13).

### Quantification of methyl salicylate

Quantification of MeSA was carried out by a previously described protocol (Schmelz *et al.*, 2004) with minor modifications. A sample of 100 mg of leaf tissue was ground in liquid nitrogen and extracted in 800 µl of extraction buffer (1-propanol:water:hydrochloric acid at a 2:1:0.005 ratio) with 10 ng of 3ʹ-methylacetophenone (internal standard). Samples were re-homogenized by adding 1 ml of dichloromethane (DCM). The DCM layer was separated by centrifugation at 12000 *g* for 30 s and collected in a 2 ml glass vial, and the sample was further concentrated to ~100 µl using inert nitrogen gas. A 1 µl aliquot of the sample was injected manually into the inlet injector port (held at a temperature of 250 °C) of a single quadrapole GC-MS system (Agilent 7890A GC and Agilent 5975-Inert XL EL/CL MSD MS). Compounds were separated on a SUPELCOWAX® 10 Capillary GC column (30 m×0.20 µm) (Sigma-Aldrich) with the column temperature initially set to 60 °C and then increased to 220 °C. Helium was used as the carrier gas with a flow rate of 1 ml min^–1^. The areas of the internal standard and MeSA were calculated by extracting the peak for *m/z*=134 and *m/z*=152, respectively (Supplementary Figs S14 and S15). The quantity of MeSA per gram of ground tissue was calculated from the unit area obtained for the internal standard.

### Yeast one-hybrid assay

The coding sequence of *St*ARF10 (PGSC0003DMT400020874) and the promoter sequences of *StGH3.6* (PGSC0003DMT400049613) (~2.4 kb upstream) and *AtGH3.5* (AT4G27260) (~3.0 kb upstream) were cloned into the pGEM-T Easy vector (Promega). All the constructs for the yeast one-hybrid (Y1-H) assay were generated by Gateway cloning technology (Thermo Fisher Scientific) (Deplancke *et al.*, 2004). For preparation of bait expression vectors, promoters were transferred to the destination vector pMW#2 (Addgene) through the donor vector pDONRP4-P1r. Furthermore, the yeast strain YM4271 was transformed with bait expression vectors and selected in SD -His (Synthetic dropout without histidine) medium. The prey expression vector was prepared by transferring the coding sequence of *St*ARF10 to the destination vector pDEST-2µ-Gal4-AD via the donor vector pDONR221. The yeast strain Yα1867 was transformed with the prey expression vector and selected in SD -Trp (Synthetic dropout without tryptophan) medium. To study the interaction between the promoters and *St*ARF10, the prey yeast (Yα1867-*St*ARF10) and either of the bait yeasts (YM4271-prom-*St*GH3.6 or YM4271-prom-*At*GH3.5) were mated by mixing in a 1:1 ratio and allowed to grow in YPDA medium. The mated yeast clones were then selected on SD -His, -Trp medium. The interaction was further confirmed by growing the mated yeast clones on SD -His, -Trp medium supplemented with increasing concentrations of 3-amino-1,2,4-triazole (3-AT).

### Electrophoretic mobility shift assay

For *St*ARF10-6×His protein preparation, the coding sequence of *St*ARF10 was PCR amplified and cloned into the pET28a^+^ vector (Novagen). Protein expression was performed using *Escherichia coli* BL21(DE3) cells as host, followed by Ni-NTA affinity column-based protein purification. For bait DNA preparation, promoter fragments P1, P2, and P3 of prom:*St*GH3.6 were PCR amplified from potato genomic DNA, and promoter fragment P4 of prom:*At*GH3.5 was PCR amplified from Arabidopsis genomic DNA. All the fragments were cloned in the pGEM-T Easy sub-cloning vector (Promega) and their sequences were verified. For the electrophoretic mobility shift assay (EMSA), probes were prepared by labeling promoter fragments with γ-^32^P-ATP using a KinaseMax End-Labeling kit (Ambion). The binding reactions were carried out as described previously (Chen *et al.*, 2004).

### Primer information

Details of all primers are provided in Supplementary Table S2.

### Accession numbers

*GAPDH*: PGSC0003DMT400044944, *StARF10:* PGSC0003 DMT400020874, *StARF16:* PGSC0003DMT400062489, *U6:* X60506, *StPR1:* AY050221, *StYUCCA1:* PGSC0003DMT400067103, *StLAX4:* PGSC0003DMT400049377, *StTIR1:* PGSC0003DMT400029517, *StIAA16:* PGSC0003DMT400050101, *StGH3*.*6*: PGSC0003DMT 400049613, *StNPR1:* XM_006357647, *StMES1:* PGSC0003DMT 400019806, *StBSMT1:* XM_006354611.

## Results

### Potato miRNAs exhibit altered expression upon *P. infestans* infection

To decipher the role of miRNAs in the potato–*P. infestans* interaction, 10 miRNAs (miR156, miR159, miR160, miR166, miR169, miR171, miR172, miR396, miR414, and miR1533) were shortlisted based on previous reports of their predicted role in biotic interactions in various plants (Bazzini *et al.*, 2007; Lu *et al.*, 2007; Xin *et al.*, 2010; Guo *et al.*, 2011; Amin *et al.*, 2011; Lang *et al.*, 2011). WT *S. chacoense*, a susceptible variety widely employed in Indian potato-breeding projects, was used for infection studies with *P. infestans*. While all 10 miRNAs were detected in *S. chacoense* (Supplementary Fig. S2A), differential expression upon infection was observed for only five miRNAs (miR159, miR160, miR166, miR172, and miR396) (Supplementary Fig. S2B–G). Although all these miRNAs were promising candidates, the most interesting were miR159, miR160, and miR166, because they showed significant up-regulation as early as 24 hpi. We chose to characterize miR160 in detail as it is known to play an important role in auxin signaling (Mallory *et al.*, 2005; Turner *et al.*, 2013) and auxin has been shown to be associated with SAR responses (Truman *et al.*, 2010).

### Expression of miR160 and its target *StARF10* are altered in both local and systemic leaves upon infection

**Fig. 1.**
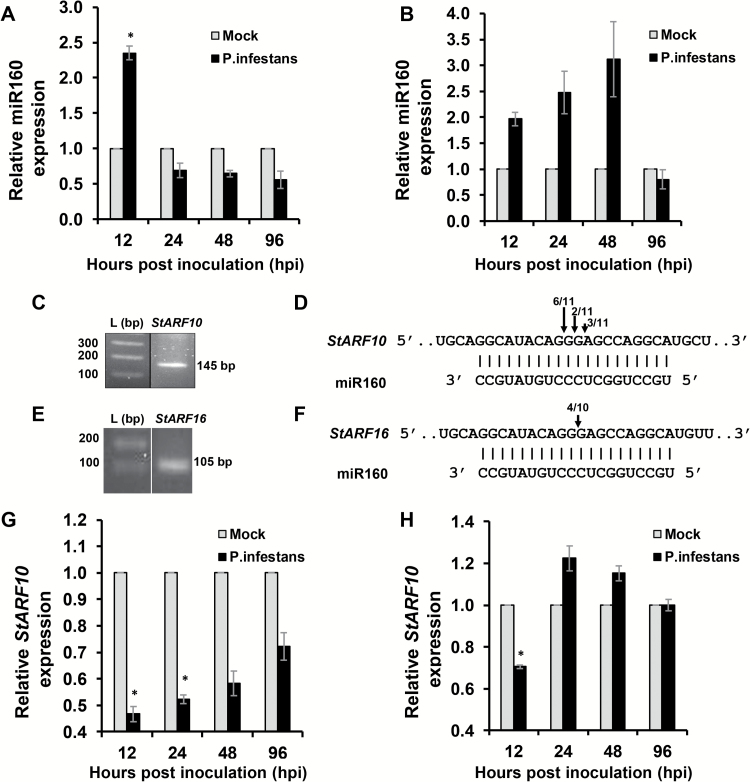
Expression of miR160 and *StARF10* in local and systemic leaves of wild-type potato (*Solanum chacoense*) upon *Phytophthora infestans* infection. (A, B) qRT–PCR analysis of miR160 levels in local leaves (A) and systemic leaves (B) at different time points post-infection. (C–F) RLM-RACE-based isolation of miR160 cleavage product of *StARF10* (C) and *StARF16* (E); partial mRNA sequence of *StARF10* (D) and *StARF16* (F) aligned with miR160. Numbers denote the fraction of cloned cleavage products that terminated at different positions (denoted by arrows). (G, H) qRT–PCR analysis of *StARF10* in local leaves (G) and systemic leaves (H) at different time points post-infection. For miR160 and *StARF10* expression analysis, data represent the mean±SE of two biological replicates with three technical replicates each, plotted by normalizing the *P. infestans*-treated values with mock-treated values for each time point. Asterisks indicate significant differences (*P*<0.05) between *P. infestans*-treated and mock-treated samples for the respective time point as analyzed by Student’s *t*-test.

To analyze the involvement of miR160 in local defense and SAR responses, expression analysis was performed in local and systemic leaves of potato after *P. infestans* infection. In local leaves, miR160 levels were significantly enhanced at 12 hpi, followed by a decrease at later time points (Fig. 1A). However, in systemic leaves, miR160 levels constantly increased from 12 to 48 hpi, followed by a decrease at 96 hpi (Fig. 1B). We observed a corresponding increase in the accumulation of miR160 (~5–6-fold) in the phloem-enriched exudates of potato at 12 hpi (Supplementary Fig. S3).

Using target prediction software, we predicted six targets of miR160 in potato: five were auxin response factors (*StARF10*, *StARF10-2*, *StARF16*, *StARF16-2*, and *StARF17*) and one was a MAP kinase (*StMAPK9*) (Supplementary Table S1). Of these, only *StARF10* (Fig. 1C, D) and *StARF16* (Fig. 1E, F) were validated as true targets of miR160 through cleavage site mapping assay. *StARF10* appeared to be the stronger target, as it had a higher cleavage frequency (11/11) than *StARF16* (4/10) (Fig. 1D, F). To investigate the effect of *P. infestans* infection on targets of miR160, expression of *StARF10* was analyzed in local and systemic leaves of *S. chacoense*. qRT–PCR analysis indicated an overall reduction in expression of *StARF10* in local leaves compared with mock-treated plants (Fig. 1G). However, in *P. infestans*-treated plants, a steady increase in *StARF10* expression was also observed over time (from 12 to 96 hpi) (Fig. 1G). In systemic leaves, *StARF10* expression was reduced significantly at 12 hpi, followed by an increase at 24 and 48 hpi and a decrease at 96 hpi (Fig. 1H). Comparison of miR160 and *StARF10* levels suggested a strong antagonistic relationship, specifically at 12 hpi, in both local (Fig. 1A, G) and systemic (Fig. 1B, H) leaves (Supplementary Fig. S4B). Overall, our findings suggested a possible role for miR160 and *StARF10* in local defense and SAR responses of potato against *P. infestans*.

### Both overexpression and knockdown of miR160 result in enhanced susceptibility

**Fig. 2.**
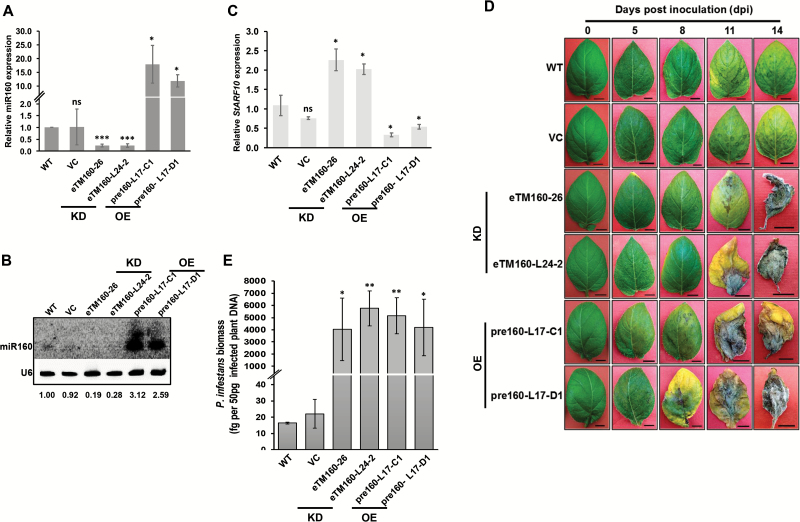
miR160 knockdown (KD) and overexpression (OE) lines are highly susceptible to *P. infestans*. (A, B) Relative levels of miR160 in two different KD (eTM160-26 and eTM160-L24-2) and OE (pre160-L17-C1 and pre160-L17-D1) lines, quantified by qRT–PCR (A) and northern blot analysis (B). (C) qRT–PCR analysis of *StARF10* in miR160 KD and OE lines. (D) Late blight progression in wild-type (WT), vector control (VC; transformed with empty pBI121 vector), and miR160 KD and OE plants monitored for a period of 14 days. Scale bars=1 cm. (E) qRT–PCR analysis of *P. infestans* genomic DNA from infected plants at 14 days post-inoculation.

To understand the detailed function of miR160 in the potato–*P. infestans* interaction, OE and KD lines of miR160 were raised in potato (Supplementary Fig. S5). As *S. chacoense* is not amenable to transformation, *S. tuberosum* cv. Désirée—a susceptible variety—was used for the generation of transgenic lines. Based on the expression patterns of *St-pre160* (the precursor of miR160), miR160, and *StARF10* (Supplementary Fig. S5A, C), the OE lines pre160-L17-C1 and pre160-L17-D1 were selected for further analysis (Fig. 2A–C). Similarly, the KD lines eTM160-L24-2 and eTM160-26 were selected on the basis of the expression patterns of eTM160 (target mimic), miR160, and *StARF10* (Supplementary Fig. S5B, D; Fig. 2A–C). No drastic morphological changes were observed in any of the miR160 OE and KD lines (Supplementary Fig. S6), indicating that their growth was comparable to that of WT plants. To assess the function of miR160 in the local defense response of potato, miR160 OE and KD lines were challenged with *P. infestans* and disease progression was monitored for a period of 14 days. Both miR160 OE and KD lines started developing early symptoms of infection at around 8 dpi, compared with 11 dpi in WT and vector control (VC) plants (Fig. 2D). By 14 dpi, most of the OE and KD lines exhibited severe disease symptoms compared with WT and VC plants (Fig. 2D; Supplementary Fig. S7). The *P. infestans* load was also significantly higher in these lines at 14 dpi (Fig. 2E). These findings suggest that both OE and KD of miR160 in potato results in enhanced susceptibility to *P. infestans.*

### Increased susceptibility of miR160 transgenic lines may be related to auxin–salicylic acid antagonism

**Fig. 3.**
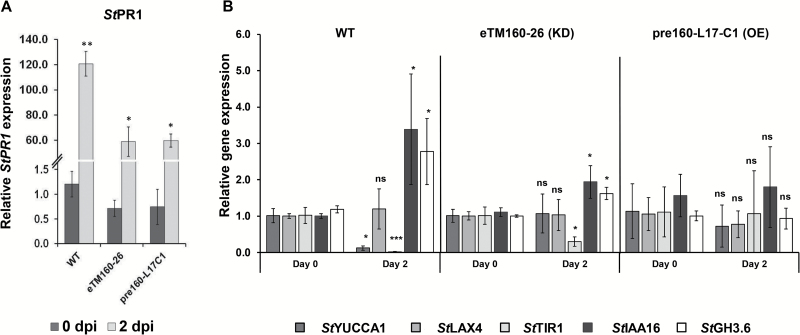
miR160 transgenic lines have altered expression of *StPR1* and auxin pathway genes. (A) *StPR1* expression after *P. infestans* infection. (B) Effect of *P. infestans* infection on expression of the auxin pathway genes *StYUCC1*, *StLAX4*, *StTIR1*, *StIAA16*, and *StGH3.6* in WT and miR160 OE and KD plants. All data represent the mean±SD of three biological replicates with three technical replicates each. Analysis was carried out for each plant type separately by comparing expression of each gene at 2 days post-inoculation (dpi) with 0 dpi. *P<0.05, **P<0.01, ***P<0.005; ns, not significant (Student’s *t*-test).

Auxin plays a critical role in plant defense (Wang *et al.*, 2007; Truman *et al.*, 2010) and antagonistic cross-talk between auxin and SA is crucial for mounting an effective defense response in plants (Huot *et al.*, 2014). In other words, to mount an SA-dependent defense response during an infection, it is important for plants to attenuate the auxin signaling pathway. miR160 is a well-known player in the auxin pathway (Mallory *et al.*, 2005; Hendelman *et al.*, 2012; Turner *et al.*, 2013; Huang *et al.*, 2016). Hence, we investigated the enhanced susceptibility phenotype of miR160 OE and KD lines by analyzing the expression of various auxin pathway genes as well as the SA pathway gene *StPR1*. Upon *P. infestans* infection, the expression of *StPR1* was induced in WT plants as well as in miR160 OE (pre160-L17-C1) and KD (eTM160-26) lines, however, the magnitude of induction was greater in WT plants (Fig. 3A). In infected WT plants, the expression of the positive auxin pathway regulators *StYUCCA1* (auxin biosynthesis gene) and *StTIR1* (*TRANSPORT INHIBITOR RESPONSE 1*, an auxin receptor) was significantly reduced relative to 0 dpi WT plants (Fig. 3B), whereas the levels of the negative regulators *StIAA16* (*INDOLE ACETIC ACID INDUCED PROTEIN 16*, and auxin signaling repressor) and *StGH3.6* (*GRETCHEN HAGEN 3.6*, an auxin/salicylic acid adenylating enzyme) were significantly elevated relative to 0 dpi WT plants (Fig. 3B). Expression of *StLAX4* (*LIKE AUXIN RESISTANT 4*, an auxin influx carrier) remained unchanged in WT plants (Fig. 3B). The KD line eTM160-26 showed lower levels of *StTIR1* and higher expression of *StIAA16* and *StGH3.6* upon infection relative to 0 dpi eTM160-26 plants (Fig. 3B); levels of *StYUCCA1* and *StLAX4* were not affected in this line. Although WT and eTM60-26 plants showed similar expression patterns for *StTIR1*, *StIAA16*, and *StGH3.6*, WT plants showed a greater magnitude of change in the expression of these genes. For *StTIR1*, we observed a ~59-fold decrease in WT plants and a ~3-fold decrease in KD lines. *StIAA16* showed a ~3.4-fold and 1.75-fold increase in WT and KD lines, respectively; for *StGH3.6*, WT and KD lines showed a ~2.3-fold and 1.6-fold increase in expression, respectively. No significant changes in expression were observed for any of these genes in the OE line (pre160-L17-C1) upon infection with *P. infestans* (Fig. 3B). These results revealed that OE or KD of miR160 leads to an imbalance in auxin–SA antagonistic cross-talk, which might result in the enhanced susceptibility phenotype.

### miR160 knockdown, but not overexpression, leads to compromised SAR

**Fig. 4.**
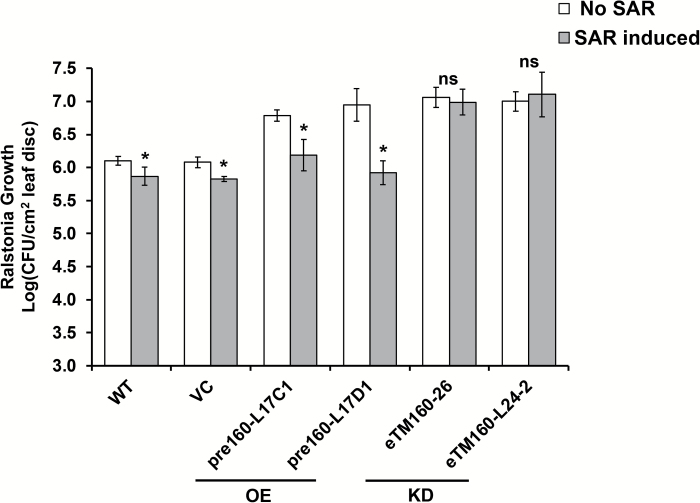
SAR response of miR160 KD and OE lines. miR160 KD lines showed a compromised SAR response. OE lines were able to induce a significant SAR response similar to that of wild-type (WT) and vector control (VC) plants, as evidenced by the reduced growth of *Ralstonia solanacearum*. Data represent the mean±SD of at least three biological replicates with three technical replicates each. ‘No SAR’ represents primary treatment with sterile water and ‘SAR induced’ represents primary treatment with *P. infestans.* Asterisks indicate values that were significantly different from the No SAR plants for each plant type (*P*<0.05; Student’s *t*-test). ns, not significant.

**Fig. 5.**
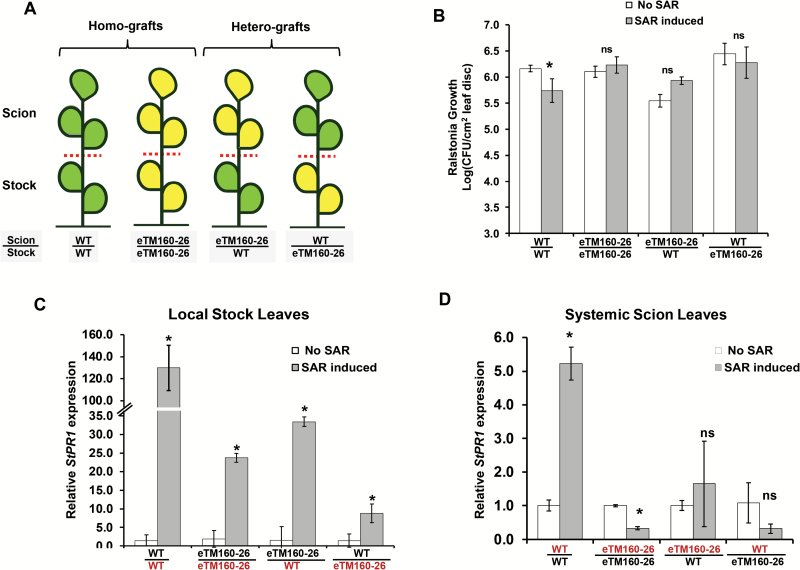
miR160 is required in both local and systemic leaves to induce an SAR response. (A) Schematics of grafts developed using WT plants and the miR160 KD line eTM160-26: homografts (WT/WT and eTM160-26/eTM160-26) and heterografts (WT/eTM160-26 and eTM160-26/WT). (B) SAR responses of the homografts and heterografts. All SAR data represent the mean±SD of at least three biological replicates with three technical replicates each. (C, D) qRT–PCR analysis of *StPR1* expression in the inoculated local stock leaves (C) and non-inoculated systemic scion leaves (D) of all the grafted plants after 4 days of primary treatment. Data represent the mean±SD of two biological replicates with three technical replicates each. ‘No SAR’ represents primary treatment with sterile water and ‘SAR induced’ represents primary treatment with *P. infestans.* Asterisks indicate values that were significantly different (*P*<0.05; Student’s *t*-test) from the No SAR plants for each plant type or graft type. ns, not significant.

To understand the function of miR160 in the SAR of potato, the miR160 OE and KD lines were assessed for the effectiveness of their SAR response. Interestingly, the miR160 KD lines failed to mount an effective SAR, whereas the OE lines exhibited a significant SAR similar to that observed in WT and VC plants (Fig. 4). To understand the compromised SAR response of miR160 KD lines, we investigated whether the SAR defect was associated with the local or systemic leaves of KD lines. SAR assays were performed on homografts (WT/WT and eTM160-26/eTM160-26) and heterografts (eTM160-26/WT and WT/eTM160-26) generated with WT plants and the KD line eTM160-26 (Fig. 5A). The stocks of the grafted plants were treated with either sterile water or *P. infestans*, and the scions were treated with *R. solanacearum*. Consistent with our previous results (Fig. 4), WT/WT homografts showed significant SAR development and KD homografts (eTM160-26/eTM160-26) did not exhibit an SAR (Fig. 5B). This suggested that the grafting process had not affected the SAR response. Interestingly, neither of the heterograft types demonstrated a significant SAR, suggesting that KD of miR160 renders both local and systemic leaves defective in SAR. Consistent with this, the expression of the SAR marker gene *StPR1* was induced in local stock leaves of all grafted plants, with the highest magnitude of induction in WT/WT homografts (Fig. 5C). In contrast, in systemic scion leaves, *StPR1* expression was induced only in WT/WT grafts (Fig. 5D), reflecting the compromised SAR in the other grafts. Overall, these results indicated that miR160 is required in both local and systemic leaves for effective SAR development.

### Salicylic acid and methyl salicylate levels are reduced in miR160 KD lines

**Fig. 6.**
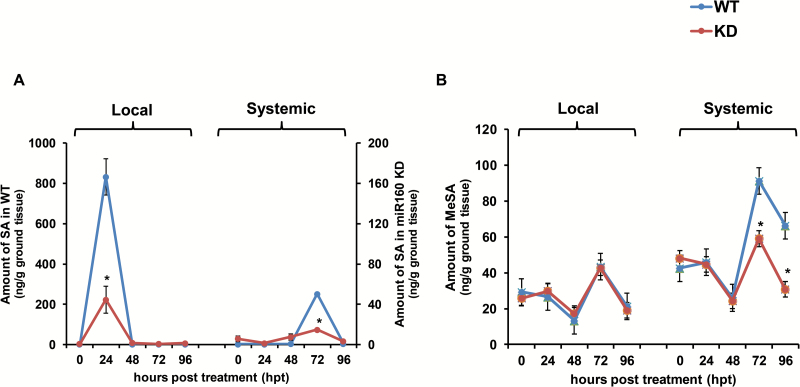
Analysis of salicylic acid (SA) and methyl salicylate (MeSA) levels in WT and eTM160-26 KD lines after arachidonic acid (AA) treatment. (A) HR-MS-based analysis of SA accumulation and (B) GC-MS-based analysis of MeSA accumulation in local and systemic leaves of WT and eTM160-26 KD lines at different time points after treatment with 0.05 mM AA. Samples were analyzed at 0, 24, 48, 72, and 96 hours post-treatment in local (AA-treated) and systemic (AA-untreated) leaves. Data represent the mean±SD of three biological replicates with three technical replicates each. Asterisks indicate statistically significant differences (*P*<0.05; Student’s *t*-test). ns, not significant.

To investigate the SAR-defective phenotype of the miR160 KD lines, the SAR-associated signals SA and MeSA were measured in local and systemic leaves of miR160 KD line eTM160-26 and WT plants after AA treatment. AA, a PAMP of *P. infestans*, has been used in previous studies of the SAR in potato because of its potential to trigger an effective SAR response (Coquoz *et al.*, 1995; Yu *et al.*, 1997). In our analysis, the amount of SA peaked in local leaves at 24 hpt and in systemic leaves at 72 hpt for both WT and eTM160-26 plants (Fig. 6A). However, SA levels were lower in the KD line compared with the WT plants at these time points (Fig. 6A). This observation suggested reduced SA signaling in both local and systemic leaves of miR160 KD plants. Levels of MeSA, the only known mobile SAR signal in potato (Manosalva *et al.*, 2010), did not differ in local leaves of WT and miR160 KD plants (Fig. 6B). However, MeSA levels were lower in systemic leaves of the KD line at 72 and 96 hpt compared with the levels in WT plants (Fig. 6B). This analysis indicated that KD of miR160 affects SA levels in both local and systemic leaves, and MeSA levels in systemic leaves, of potato.

### SAR pathway genes are affected in miR160 KD lines

**Fig. 7.**
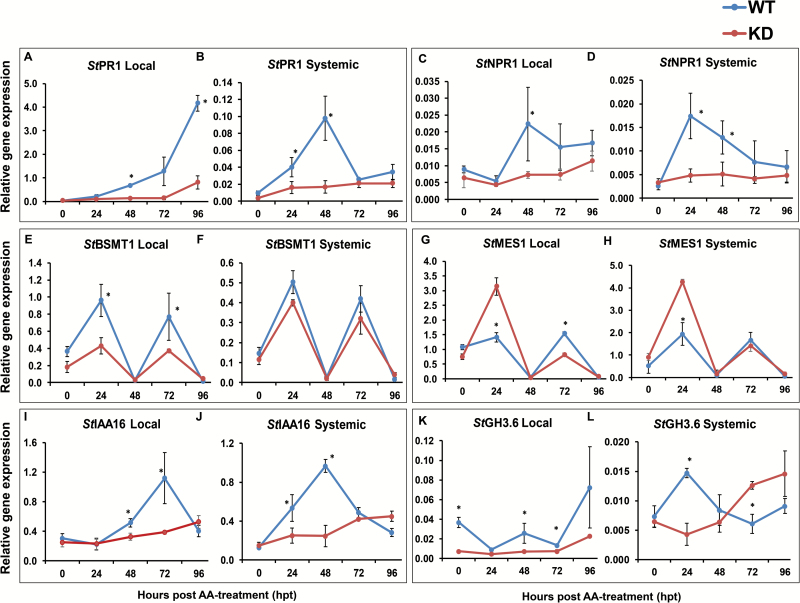
Multiple SAR-associated genes are affected in miR160 KD lines. (A, B) *StPR1* expression in local (A) and systemic (B) leaves. (C, D) *StNPR1* expression in local (C) and systemic (D) leaves. (E, F) *StBSMT1* expression in local (E) and systemic (F) leaves. (G, H) *StMES1* expression in local (G) and systemic (H) leaves. (I, J) *StIAA16* expression in local (I) and systemic (J) leaves. (K, L) *StGH3.6* expression in local (K) and systemic (L) leaves. All data represent the mean±SD of three biological replicates with three technical replicates each. *P<0.05 (Student’s *t*-test). ns, not significant.

We also performed expression analysis of some important SAR and auxin pathway genes in local and systemic leaves of WT and miR160 KD (eTM160-26) plants. After AA treatment, the expression of *StPR1* increased in both local and systemic leaves of WT plants (Fig. 7A, B). An equivalent increase in *StPR1* expression was not observed in miR160 KD plants (Fig. 7A, B). Similarly, the expression of *StNPR1* (*NONEXPRESSOR OF PR1*), the master regulator of SAR, was also induced in local and systemic leaves of WT plants, whereas the miR160 KD line failed to exhibit a similar increase in *StNPR1* expression (Fig. 7C, D). Furthermore, *StBSMT1* (*BENZOIC ACID/SA CARBOXYL METHYL TRANSFERASE 1*), the gene involved in the conversion of SA to MeSA in local leaves, exhibited an oscillating expression pattern, with peaks at 24 and 72 hpt in both local and systemic leaves of WT and KD plants (Fig. 7E, F). The magnitude of *StBSMT1* expression was reduced in local leaves of miR160 KD plants relative to WT; however, no significant changes were observed in systemic leaves (Fig. 7E, F). *StMES1* (*METHYL ESTERASE 1*), the gene involved in the conversion of MeSA to SA in systemic leaves, also exhibited an oscillating expression pattern similar to that of *StBSMT1*, but *StMES1* expression was higher in both local and systemic leaves of miR160 KD lines at 24 hpt (Fig. 7G, H). Additionally, the expression of the auxin signaling repressor *StIAA16* increased in both local and systemic leaves of WT plants after AA treatment; a similar increase of *StIAA16* expression was not observed in miR160 KD plants (Fig. 7I, J). *StGH3.6*, a potential auxin and SA conjugator in potato, had lower expression in local leaves of miR160 KD plants, relative to WT, at all time points (Fig. 7K). However, in systemic leaves of the KD line, its levels were low at 24 hpt and high at 72 and 96 hpt (Fig. 7L). Unlike all the other genes tested, the expression pattern of *StGH3.6* in systemic leaves of the KD plants was opposite to that of WT plants, suggesting a possible dysregulation of this gene specifically in systemic leaves of miR160 KD plants. These results indicate that miR160 KD plants failed to induce the negative regulators of auxin signaling, which could potentially lead to unsuccessful attenuation of the auxin pathway. Taken together, these analyses reveal dysregulation of several genes involved in the SAR pathway associated with miR160 KD, and provide further evidence that auxin–SA antagonistic cross-talk appears to play role in the SAR responses of potato.

### *StGH3*.6, a mediator of auxin–SA cross-talk, is regulated by *St*ARF10

**Fig. 8.**
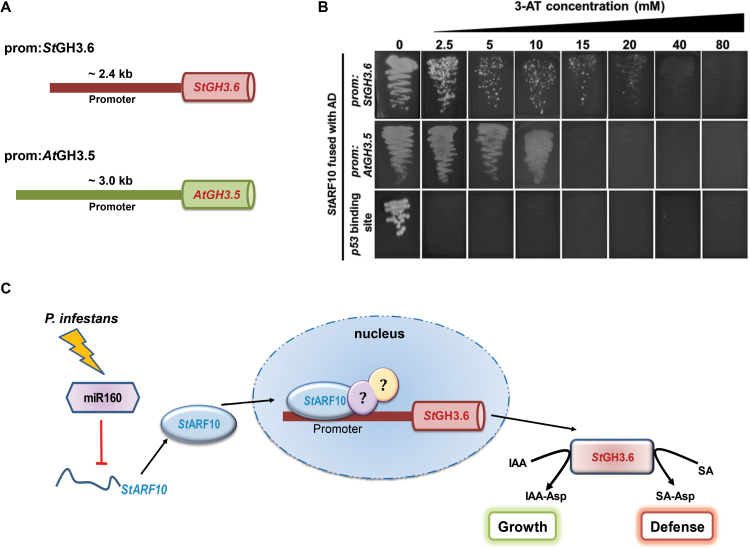
*St*ARF10 binds to the promoters of *StGH3.6* and *AtGH3.5*. (A) Diagrammatic representation of the promoters used for the yeast one-hybrid (Y1-H) assay: regions ~2.4 kb upstream of *StGH3.6* (prom:*StGH3.6*) and ~3.0 kb upstream of *AtGH3*.5 (prom:*AtGH3.5*) were used. (B) Yeast strains containing *St*ARF10 prey protein and prom:*St*GH3.6 bait grew in media containing up to 20 mM 3-amino-1,2,4-triazole (3-AT); strains containing *St*ARF10 prey and prom:*At*GH3.5 bait grew in media containing up to 10 mM 3-AT. The growth of yeast strains indicates the binding of *St*ARF10 to both *StGH3.6* and *AtGH3.5* promoters. The p53 binding site was used as a negative control; inhibition of yeast growth at all the concentrations of 3-AT suggests no interaction between the p53 binding site and *St*ARF10 protein. AD, activation domain. (C) A proposed model showing the control of *StGH3.6* by a miR160-*St*ARF10 module. Upon infection by *P. infestans*, changes in the expression of miR160 affect the levels of *StARF10* mRNA. This in turn affects the levels of *St*ARF10 protein that could enter the nucleus. In the nucleus, *St*ARF10 protein binds to the promoter of *StGH3.6* and affects its expression, possibly in combination with unknown partners (represented by circles containing question marks). Altered *St*GH3.6 protein levels might potentially affect the auxin–SA antagonistic cross-talk and the balance of the defense and growth pathways. This may be one of the pathways by which potato plants regulate local and SAR defense responses during *P. infestans* infection. IAA, indole-3-acetic acid; IAA-Asp, conjugated IAA–aspartic acid; SA, salicylic acid; SA-Asp, conjugated SA–aspartic acid.

As mentioned above, auxin–SA antagonistic cross-talk is important for mounting an effective defense response in plants. A recent study demonstrated the role of Arabidopsis Gretchen Hagen 3.5 (*At*GH3.5) as a mediator of this cross-talk, since it can conjugate both indole-3-acetic acid (IAA), an auxin, and SA (Westfall *et al.*, 2016). *At*GH3.5 has also been shown to be involved in local defense and SAR responses (Zhang *et al.*, 2007). In our study, we observed differential expression of *StGH3.6*, a potato homolog of *AtGH3.5* (sequence similarity of 77.6%; Supplementary Fig. S8), in miR160 OE and KD lines (Supplementary Fig. S9), as well as under infective conditions (Figs 3B and 7K, L). Previous studies have demonstrated the control of ARF transcription factors on the *GH3* family of genes (Ulmasov *et al.*, 1997; Yang *et al.*, 2006). This prompted us to hypothesize that miR160 could regulate *StGH3*.6 expression through *St*ARF10 in potato. To test this hypothesis, Y1-H analysis and EMSA were performed using *St*ARF10 protein and the promoters of potato *StGH3.6* (prom-*StGH3.6*) and Arabidopsis *AtGH3*.5 (prom-*AtGH3.5*) (Fig. 8A; Supplementary Fig. S11A). For Y1-H assays, the interaction of *St*ARF10 protein was explored with the ~2.4 kb promoter of *StGH3.6* and the ~3.0 kb promoter of *AtGH3*.5 (Fig. 8A). Mated yeast colonies containing *St*ARF10 with the promoter of either *StGH3.6* or *AtGH3.5* grew robustly on the selection media (SD -His -Trp) with increasing concentrations of 3-AT (Fig. 8B). This suggested the binding of *St*ARF10 to both the promoter sequences. As the protein sequences of *St*ARF10 and *At*ARF10 are highly similar (70.3%; Supplementary Fig. S10), it is possible that Arabidopsis *At*ARF10 also binds to these promoter sequences. EMSA analysis further confirmed the interaction of *St*ARF10 protein with the promoter fragments of prom:*StGH3.6* and prom:*AtGH3*.5 (Supplementary Fig. S11). Together, our results suggest that a miR160-*St*ARF10 module could possibly affect *StGH3.6* expression, thereby modulating the cross-talk between auxin-mediated growth and the SA-mediated defense response during the potato–*P. infestans* interaction (Fig. 8C).

## Discussion

Several reports have demonstrated differential expression of miRNAs during a variety of plant–pathogen interactions (Bazzini *et al.*, 2007; Lu *et al.*, 2007; Xin *et al.*, 2010; Guo *et al.*, 2011; Amin *et al.*, 2011; Lang *et al.*, 2011; Zhao *et al.*, 2012; Li *et al.*, 2014). However, no study has yet explored the role of miRNAs in the potato–*P. infestans* interaction. Understanding this process could enhance our knowledge toward combating late blight disease. Additionally, while miRNAs are well demonstrated to be involved in local defenses, their role in SAR has not been explored. In this study, from a shortlist of 10 potential miRNAs, we selected miR160 and investigated its role in the defense responses of potato against *P. infestans*. By using a combination of approaches such as expression profiling, transgenic studies, infection analysis, and grafting, we show that miR160 is crucial for eliciting local defense and SAR responses during the potato–*P. infestans* interaction.

### The miR160 expression pattern is important for eliciting local defense and SAR responses in potato

miR160, a conserved miRNA among various plant species, is known to play a vital role in plant growth and development (Mallory *et al.*, 2005; Hendelman *et al.*, 2012; Turner *et al.*, 2013; Huang *et al.*, 2016); however, its role in defense was discovered only recently (Li *et al.*, 2010, 2014). Previous reports have demonstrated the induction of miR160 in local infected leaves of various plants upon bacterial or fungal infection (Xin *et al.*, 2010; Lang *et al.*, 2011; Pérez-Quintero *et al.*, 2012). Similarly, we observed the induction of miR160 expression in local leaves of potato upon *P. infestans* infection (Fig. 1A). In addition, systemic leaves also exhibited induced expression of miR160 (Fig. 1B), indicating a potential role for miR160 in both local and SAR responses of potato. The enhanced susceptibility to *P. infestans* observed in both miR160 OE and KD lines is an indication of the breakdown of local defense responses (Fig. 2D, E; Supplementary Fig. S7). Our findings in the miR160 OE lines contrasted with previous observations. In Arabidopsis, miR160 OE plants were shown to have no difference in basal resistance to bacterial infection compared with WT plants (Li *et al.*, 2010). In rice, however, miR160 OE plants exhibited increased basal resistance against *Magnaporthe oryzae* relative to WT plants (Li *et al.*, 2014). Such contradictory responses of miR160 OE plants could be due to the different types of plant–pathogen interaction studied. Furthermore, the enhanced susceptibility observed in both miR160 OE and KD lines in our study came as a surprise. Similar observations were made with a plasmodesmata localizing protein, PDLP5, in Arabidopsis. Lim *et al.* (2016) have shown that both 35S-*PDLP5* (OE) and *pdlp-5* (KD) plants show a similar phenotype of a compromised SAR response. These results indicated that it is not improbable for a similar phenotype to develop in an OE and a KD line for a given gene.

The SAR phenotype was, however, different in the miR160 OE and KD lines we studied. The miR160 KD lines failed to elicit a SAR, whereas the OE lines successfully mounted a SAR (Fig. 4). Similar to the miR160 KD lines, many SAR-deficient mutants of Arabidopsis, such as *npr1* (Cao *et al.*, 1997), *pad4* (Jirage *et al.*, 1999), and *sid2* (Wildermuth *et al.*, 2001), are affected in basal defense as well. Furthermore, the Arabidopsis mutant *eds5* is affected in basal defense but capable of mounting a SAR response, similar to our miR160 OE lines (Rogers and Ausubel, 1997). We propose that the compromised SAR response of the miR160 KD lines could be because of either or both of the following possibilities: (i) local leaves of KD lines have failed to generate and/or transport the SAR signal to systemic leaves, (ii) systemic leaves have failed to perceive and/or process the SAR signal transported from local leaves. Several previous studies have used grafting as a powerful tool to delineate questions associated with SAR responses in plants (Vernooij *et al.*, 1994; Park *et al.*, 2007). Our grafting study with WT and KD plants suggested that the miR160 KD lines were defective in both local and systemic responses (Fig. 5).

On the basis of these findings, we contend that a dynamic expression pattern of miR160 in local and systemic leaves is important for mounting local and SAR responses in potato. An initial increase of miR160 expression at 12 hpi followed by a decrease at later time points in the local leaves of WT plants (Fig. 1A) appears to be critical for the establishment of local defense responses. This notion is supported by the fact that both miR160 OE and KD plants (which constitutively overexpress or knockdown miR160 and thus cannot achieve similar dynamic expression patterns) show a breakdown of local defense (Fig. 2). In the systemic leaves of WT plants, the increased miR160 expression up to 48 hpi seems to be vital for the development of SAR (Fig. 1B). We conclude this because a compromised SAR response is observed only in miR160 KD lines and not in OE lines (Fig. 4).

### miR160 may be involved in the SA pathway

To elucidate the defects observed in the local and systemic leaves of miR160 KD lines, SAR-associated signals (SA and MeSA); (Fig. 6), as well as the expression of selected SAR pathway genes (Fig. 7), were analyzed in miR160 KD lines after treatment with AA. We observed reduced levels of SA, *StNPR1*, and *StPR1* in both local and systemic leaves of miR160 KD lines compared with WT plants (Figs 6A and 7A–D). This finding suggested that miR160 could positively regulate SA accumulation in both local and systemic leaves by some, as yet unknown, mechanism. In addition, reduced SA accumulation in the miR160 KD lines might have resulted in low levels of *StNPR1* and *StPR1*. In potato, MeSA was shown to act as a mobile SAR signal and *St*MES1 was found to be involved in the conversion of MeSA to SA in systemic leaves (Manosalva *et al.*, 2010). However, the role of *St*BSMT1, a homolog of the Arabidopsis SA to MeSA converting enzyme (Chen *et al.*, 2003), is unknown in potato. We observed reduced *StBSMT1* (Fig. 7E, F) and increased *StMES1* (Fig. 7G, H) expression in miR160 KD lines relative to WT plants; however, it is not clear how miR160 affects the expression of these genes. Although local leaves of the KD lines had low SA (Fig. 6A) and reduced *StBSMT1* expression (Fig. 7E), their accumulation of MeSA was comparable to that of WT plants (Fig. 6B). This might have resulted from any of the following possibilities: (i) the low SA levels in KD plants were perhaps sufficient to produce an optimum amount of MeSA; (ii) the conversion of SA to MeSA might have been done by other homologs of *St*BSMT1; or (iii) MeSA is not effectively transported from the local leaves of KD plants. The lower levels of MeSA observed in systemic leaves at later time points relative to WT (Fig. 6B) suggest that MeSA transport was possibly affected in the miR160 KD plants. This could be the cause of the reduced SA levels in the systemic leaves of KD plants (Fig. 6A). Taken together, our results suggest that miR160 may be involved in the SA pathway and is crucial for SAR in potato.

### Auxin–SA cross-talk and the miR160-*St*ARF10- *StGH3.6* module

For plants, the maintenance of both defense and development can be energy intensive. The antagonistic cross-talk of auxin and SA is one of the mechanisms adopted by plants to mediate trade-offs between growth and defense to mount an effective defense response (Huot *et al.*, 2014). In other words, during an infection, plants seem to redistribute their energy by attenuating auxin signaling and directing resources to trigger defense responses (Kazan and Manners, 2009). Our results suggest that miR160 is involved in this cross-talk through its target *St*ARF10. In our local infection experiments, we observed that infected WT potato plants were able to repress positive (*StYUCCA1* and *StTIR1*) and induce negative (*StIAA16* and *StGH3*.6) regulators of the auxin pathway (Fig. 3B), leading to enhanced expression of *StPR1* (Fig. 3A). However, similar attenuation of the auxin pathway (Fig. 3B) and induction of *StPR1* expression (i.e. the SA pathway; Fig. 3A) was not observed in the miR160 KD and OE plants. This observation suggested that OE and KD of miR160 affected auxin-SA cross-talk and basal defense responses in potato.

In Arabidopsis, one of the mediators of this cross-talk is Gretchen Hagen 3.5 (*At*GH3.5), an enzyme that conjugates both auxin and SA (Westfall *et al.*, 2016) and is implicated in both local defense and SAR responses (Zhang *et al.*, 2007). The molecular mechanism involved in the transcriptional control of *AtGH3.5* is not known. Here, we show that the expression of *StGH3.6*, a potato homolog of *AtGH3.5,* is affected in miR160 OE and KD lines (Figs 3B and 7K, L). Several studies have reported that ARFs can regulate some *GH3* family genes (Hagen and Guilfoyle, 2002; Staswick *et al.*, 2005), hence we wanted to understand whether *St*ARF10 (the target of miR160) could affect *StGH3.6* expression. Through Y1-H and EMSA analysis, we confirmed that the potato protein *St*ARF10 can directly bind to the promoter of both *StGH3.6* and *AtGH3.5* (Fig. 8; Supplementary Fig. S11). Further experiments could determine: (i) whether such binding has a positive or negative effect on *StGH3*.6 transcription, and (ii) whether *St*ARF10 forms dimers with itself or any other ARF partner, similar to Arabidopsis (Korasick *et al.*, 2014), to bring about this effect. Our findings provide insights regarding one of the possible mechanisms by which miR160 could be modulating auxin–SA cross-talk in potato (Fig. 8C).

Taken together, our findings demonstrate that miR160 plays a crucial role in local defense and SAR responses during the potato–*P. infestans* interaction. Our study provides new insights regarding the SAR process in potato and various genes involved in its regulation. Furthermore, we showed that miR160 is involved in antagonistic cross-talk between SA-mediated pathogen defense processes and auxin-mediated growth. The binding of the *StGH3.6* promoter by *St*ARF10 protein could be one of the mechanisms by which miR160 might modulate SA–auxin cross-talk. We speculate that the miR160-*St*ARF10 module functions differentially in local and systemic leaves to regulate *StGH3.6* expression. Our investigation has raised several novel questions, regarding whether miR160 has a role in the SAR response of other plant–pathogen interactions; whether, given the increased accumulation of miR160 in phloem-enriched exudates, it could be acting as a phloem-mobile SAR signal; and what is the role of other differentially expressed miRNAs in local and SAR responses of potato. Future investigations are required to shed light on these important questions.

## Supplementary data

Supplementary data are available at *JXB* online.

Fig. S1. Detection and confirmation of *Phytophthora infestans* and *Ralstonia solanacearum.*

Fig. S2. Detection and post-infection expression analysis of miRNAs in potato.

Fig. S3. Analysis of miR160 in phloem-enriched exudates of *S. chacoense*.

Fig. S4. Examining the inverse correlation between the expression of miR160 and *StARF10.*

Fig. S5. Confirmation of miR160 overexpression and knockdown lines in potato.

Fig. S6. Morphological phenotype of potato miR160 transgenic lines.

Fig. S7. miR160 overexpression and knockdown lines are highly susceptible to *P. infestans* infection.

Fig. S8. Sequence similarity between *At*GH3.5 and *St*GH3.6 protein sequences.

Fig. S9. Expression of *StGH3.6* in miR160 transgenic lines.

Fig. S10. Sequence similarity between *St*ARF10 and *At*ARF10 protein sequences.

Fig. S11. Electrophoretic mobility shift assay to understand the interaction of *St*ARF10 protein with promoter fragments of *StGH3.6* and *AtGH3.5*.

Fig. S12. Standard graph for absolute quantification of *P. infestans* biomass.

Fig. S13. HR-MS analysis of salicylic acid.

Fig. S14. GC-MS analysis of internal standard, 3ʹ-methylacetophenone.

Fig. S15. GC-MS analysis of methyl salicylate.

Table S1. Target prediction analysis of miR160 from potato.

Table S2. Details of the primers used in this study.

Supplementary Figures S1-S15

Supplementary Tables S1-S2
