# Supplementary material for: MiRNA160 is associated with local defense and systemic acquired resistance against Phytophthora infestans infection in potato
Source: J Exp Bot. 2018 Jan 30;69(8):2023–36. doi: 10.1093/jxb/ery025 (PMC6018911; doi:10.1093/jxb/ery025)
Supplement: Supplementary Figures S1-S15 [file ery025_suppl_supplementary_figures_s1-s15.pdf]

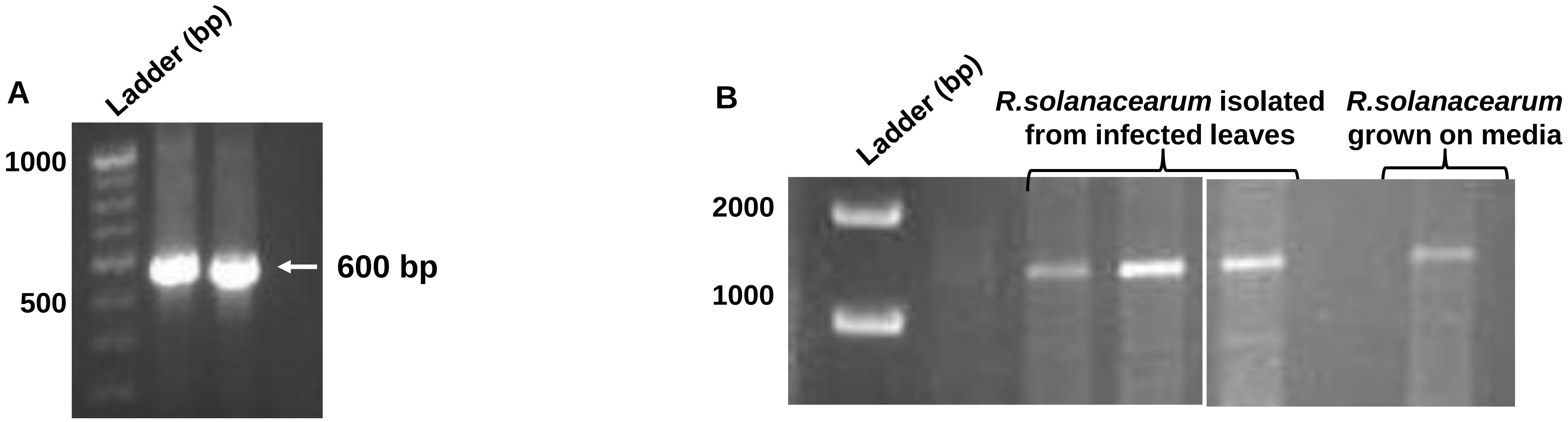

Fig. S1. Detection and confirmation of *Phytophthora infestans* and *Ralstonia solanacearum.* (A) DNA PCR of A2 Strain of *P. infestans* used in all the experiments in this study. A part of Internal Transcribed Spacer 2 (ITS2) ribosomal DNA was amplified using PINF (forward Primer) and ITS5 (reverse primer) giving a product of ~600 bp (Trout et al, 1997). (B) DNA PCR based detection of *R. solanacearum* grown on nutrient agar medium and isolated from infected potato plants. Detection was performed using primers Rs-BP4-R and Rs-BP4-L as described by Lee and Wang, (2000). Primer details are provided in Supplementary Table S2.

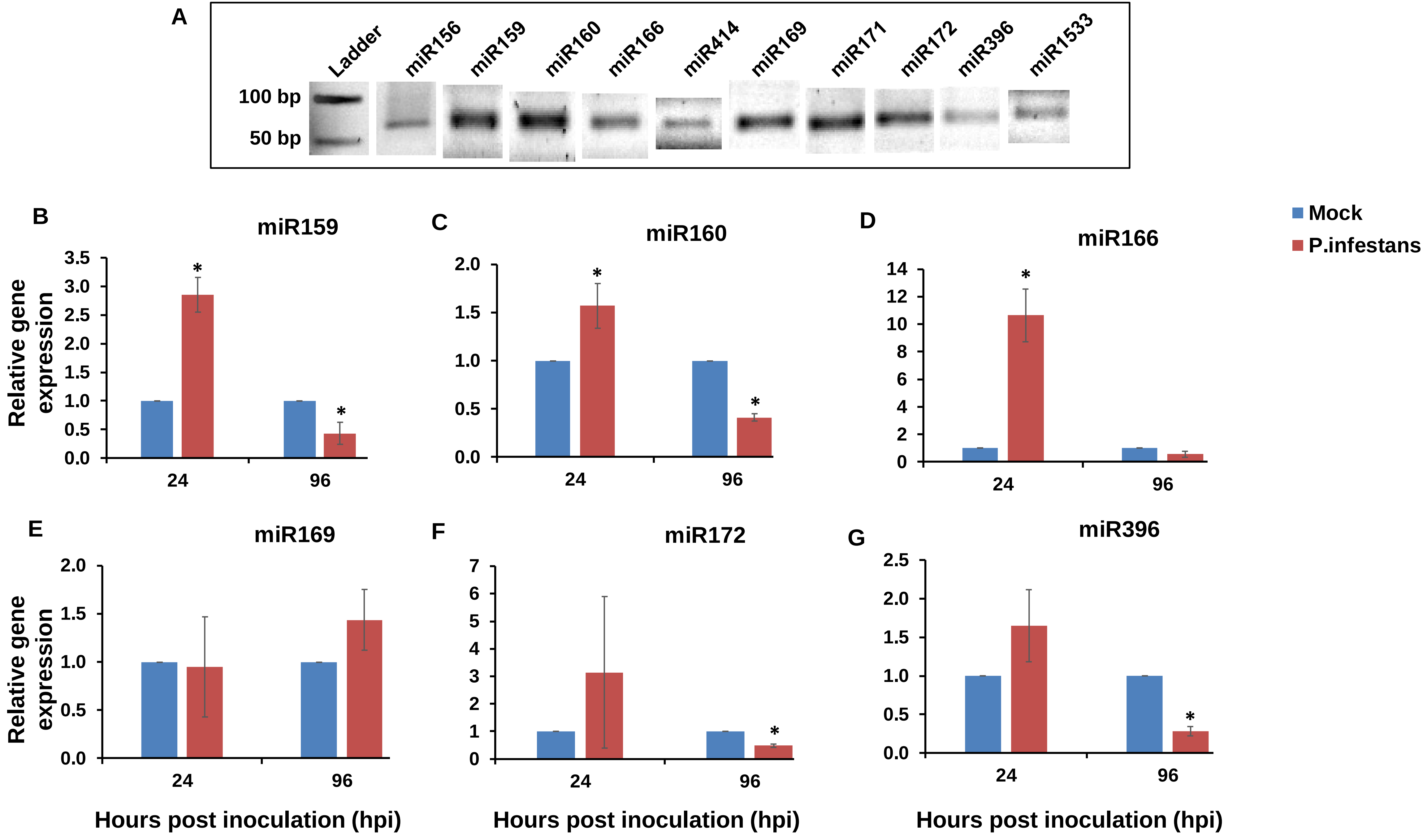
A
Ladder
miR156
miR159
miR160
miR166
miR414
miR169
miR171
miR172
miR396
miR1533
100 bp
50 bp
B
miR159
C
miR160
D
miR166
Mock
P.infestans
E
miR169
F
miR172
G
miR396
Relative gene expression
24
96
*
Hours post inoculation (hpi)

Fig. S2. Detection and post-infection expression analysis of miRNAs in potato. (A) Stem-loop RT-PCR based detection of miR156, miR159, miR160, miR166, miR169, miR171, miR172, miR396, miR414 and miR1533 in the leaf RNA of *S. chacoense*. (B-G) qRT-PCR analysis of miR159 (B), miR160 (C), miR166 (D), miR169 (E), miR172 (F) and miR396 (G) in *S. chacoense* at 24 and 96 hours post inoculation (hpi) of *P. infestans*. Data represents mean ± SE (standard error) of two biological replicates having three technical replicates each. Analysis was carried out by comparing each *P. infestans* treated sample to its corresponding mock sample of respective time-points. Asterisks indicate significant difference between the two bars being compared as per Student's t-test ($p<0.05$).

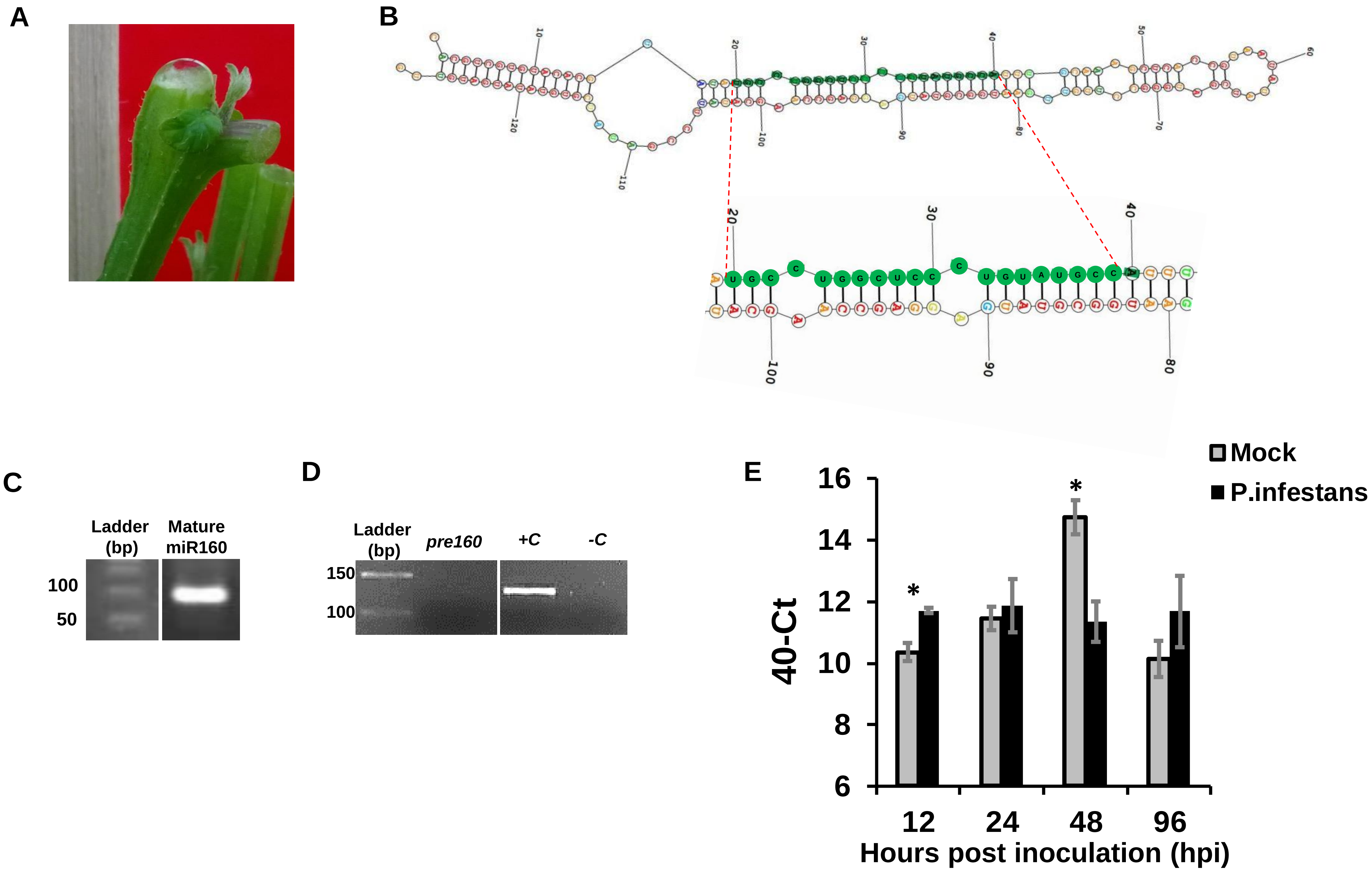
A
B
C
Ladder (bp)
Mature miR160
100
50
D
Ladder (bp)
pre160
+C
-C
150
100
E
Mock
P.infestans
40-Ct
16
14
12
10
8
6
*
*
12
24
48
96
Hours post inoculation (hpi)

Fig. S3. Analysis of miR160 in phloem enriched exudates of *S. chacoense*. (A) Phloem-enriched exudate (PEX) isolation from *S. chacoense* stem as per pervious protocol (Campbell *et al.*, 2008). (B) Stem-loop structure of precursor of potato miR160, *St-pre160.* Zoomed-in image depicts mature miR160 sequence in the stem portion of *St-pre160.* (C) Stem-Loop RT-PCR detection of mature miR160 in PEX. (D) RT-PCR detection of *St-pre160* in PEX +C is PCR positive control performed using potato leaf RNA; –C is no template control. (E) qRT-PCR analysis of mature miR160 accumulation in PEX at different time-points after *P. infestans* inoculation*.* Data has been plotted as 'total cycle number' minus 'cycle threshold' (40-Ct) values as described previously (Pant *et al.*, 2008) and represents mean ± SE of three biological replicates having three technical replicates each. Analysis was carried out by comparing each *P. infestans* treated sample to its corresponding mock sample of respective time-points. Asterisks indicate significant difference between the two bars being compared as per Student's t-test ($p < 0.05$).

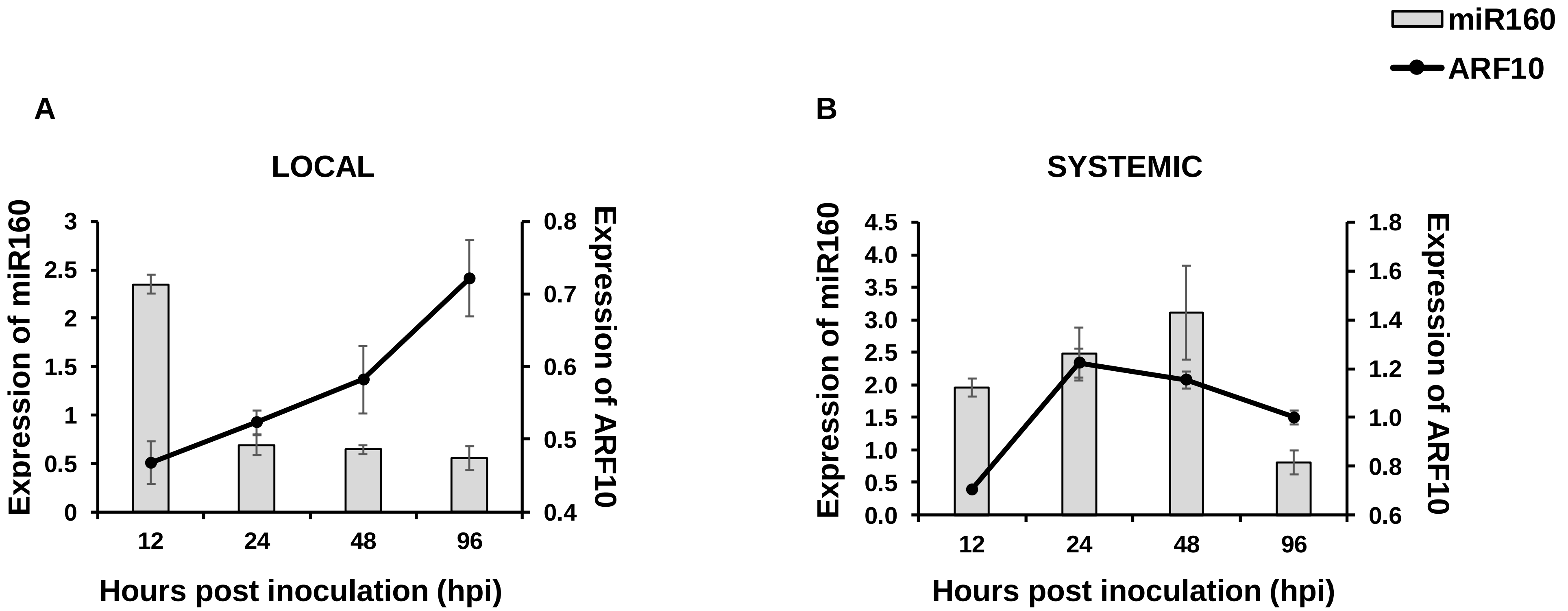

Fig. S4. Examining the inverse correlation between the expression of miR160 and *StARF10.* (A) Comparison of miR160 and *StARF10* expression levels at different time-points in local leaves. (B) Comparison of miR160 and *StARF10* expression levels at different time-points in systemic leaves. All the qRT-PCR values of miR160 and *StARF10* expression are the same as that plotted in Fig. 1. *StARF10* is plotted in secondary y-axis for clarity.

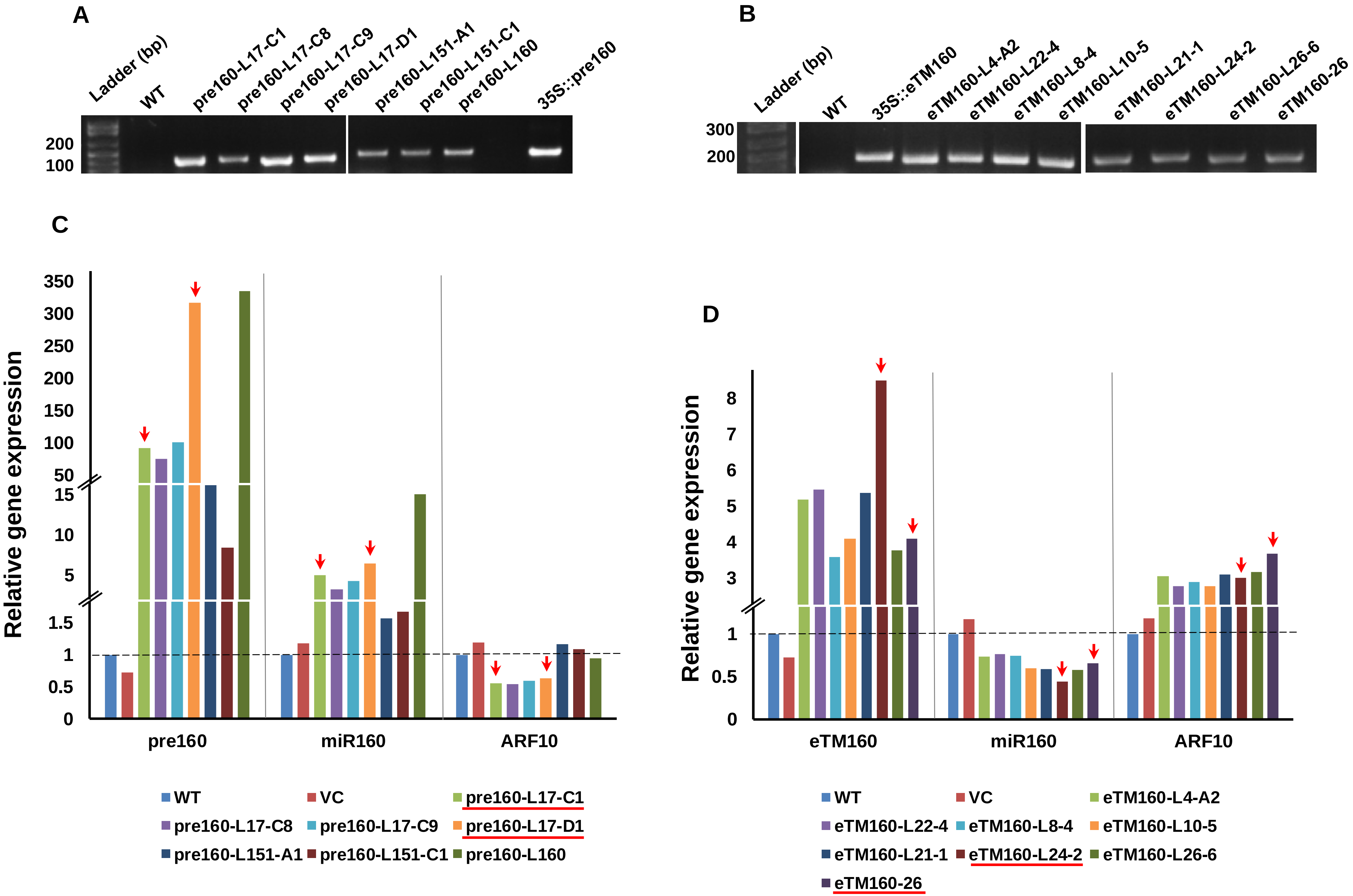
A
Ladder (bp)
WT
pre160-L17-C1
pre160-L17-C8
pre160-L17-C9
pre160-L17-D1
pre160-L151-A1
pre160-L151-C1
pre160-L160
35S::pre160
200
100
B
Ladder (bp)
WT
35S::eTM160
eTM160-L4-A2
eTM160-L22-4
eTM160-L8-4
eTM160-L10-5
eTM160-L21-1
eTM160-L24-2
eTM160-L26-6
eTM160-26
300
200
C
Relative gene expression
350
300
250
200
150
100
50
15
10
5
1.5
1
0.5
0
pre160
miR160
ARF10
WT
VC
pre160-L17-C1
pre160-L17-C8
pre160-L17-C9
pre160-L17-D1
pre160-L151-A1
pre160-L151-C1
pre160-L160
D
Relative gene expression
8
7
6
5
4
3
1
0.5
0
eTM160
miR160
ARF10
WT
VC
eTM160-L4-A2
eTM160-L22-4
eTM160-L8-4
eTM160-L10-5
eTM160-L21-1
eTM160-L24-2
eTM160-L26-6
eTM160-26

Fig. S5. Confirmation of miR160 overexpression (OE) and knockdown (KD) lines in potato. (A) Confirmation of multiple OE lines by performing genomic DNA PCR using primers pre160-FP and NOS-T-RP. The genomic DNA from wild type (WT) plants was used as negative control and the plasmid, 35S::St-pre160-pBI121, was used as positive control in the reaction. (B) Confirmation of multiple KD lines by performing genomic DNA PCR using primers eTM160-FP and eTM160-RP. The genomic DNA from wild type (WT) plants was used as negative control and the plasmid, 35S::eTM160-pCAMBIA1300, was used as positive control in the reaction. (C) qRT-PCR based analysis *St-pre160*, miR160 and *StARF10* levels in the OE lines. The OE lines, pre160-L17-C1 and pre160-L17D1 (underlined in red and red arrows), were selected for further analysis based on their high-expression of *St-pre160* and miR160 as well as reduced expression of the target *StARF10*. The data is mean of one biological replicate from three technical replicates. (D) qRT-PCR based analysis eTM160, miR160 and *StARF10* levels in the KD lines. The KD lines, eTM160-L24-2 and eTM160-26 (underlined in red and red arrows), were selected for further analysis based on their high-expression of eTM160 and the target *StARF10* as well as reduced expression of miR160. The data is mean of one biological replicate from three technical replicates. VC indicates vector control plants.

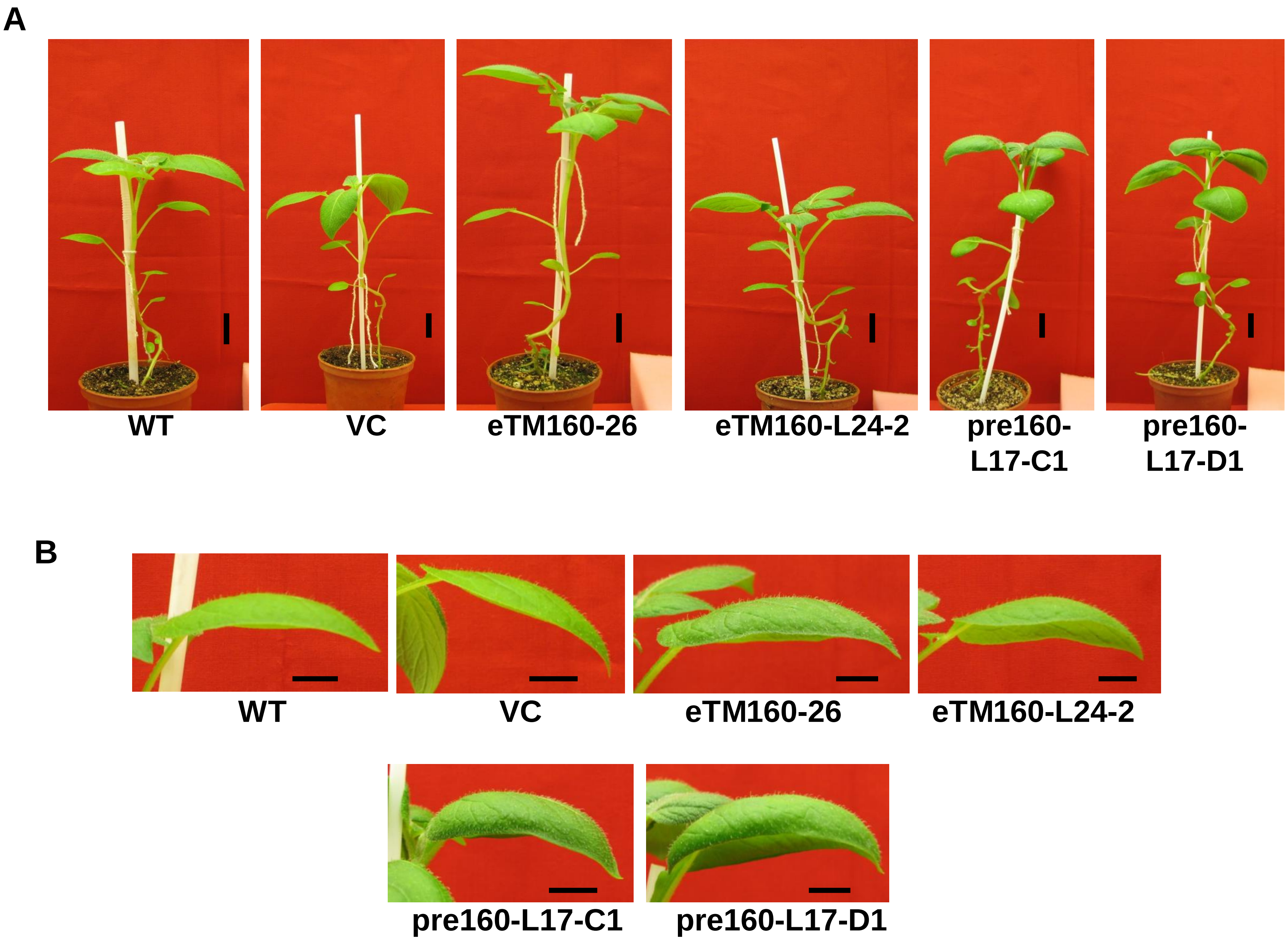
A
WT
VC
eTM160-26
eTM160-L24-2
pre160-L17-C1
pre160-L17-D1
B
WT
VC
eTM160-26
eTM160-L24-2
pre160-L17-C1
pre160-L17-D1

Fig. S6. Morphological phenotype of potato miR160 transgenic lines. (A) Morphology of the potato plants were not severely affected due to miR160 OE and KD. Black bar represents scale of 2 cm. (B) Downward curled leafs were observed only in the miR160 OE lines (pre160-L17-C1 and pre160-L17-D1). Scale bar: 1 cm. WT indicates wild-type plants, VC indicates vector control plants.

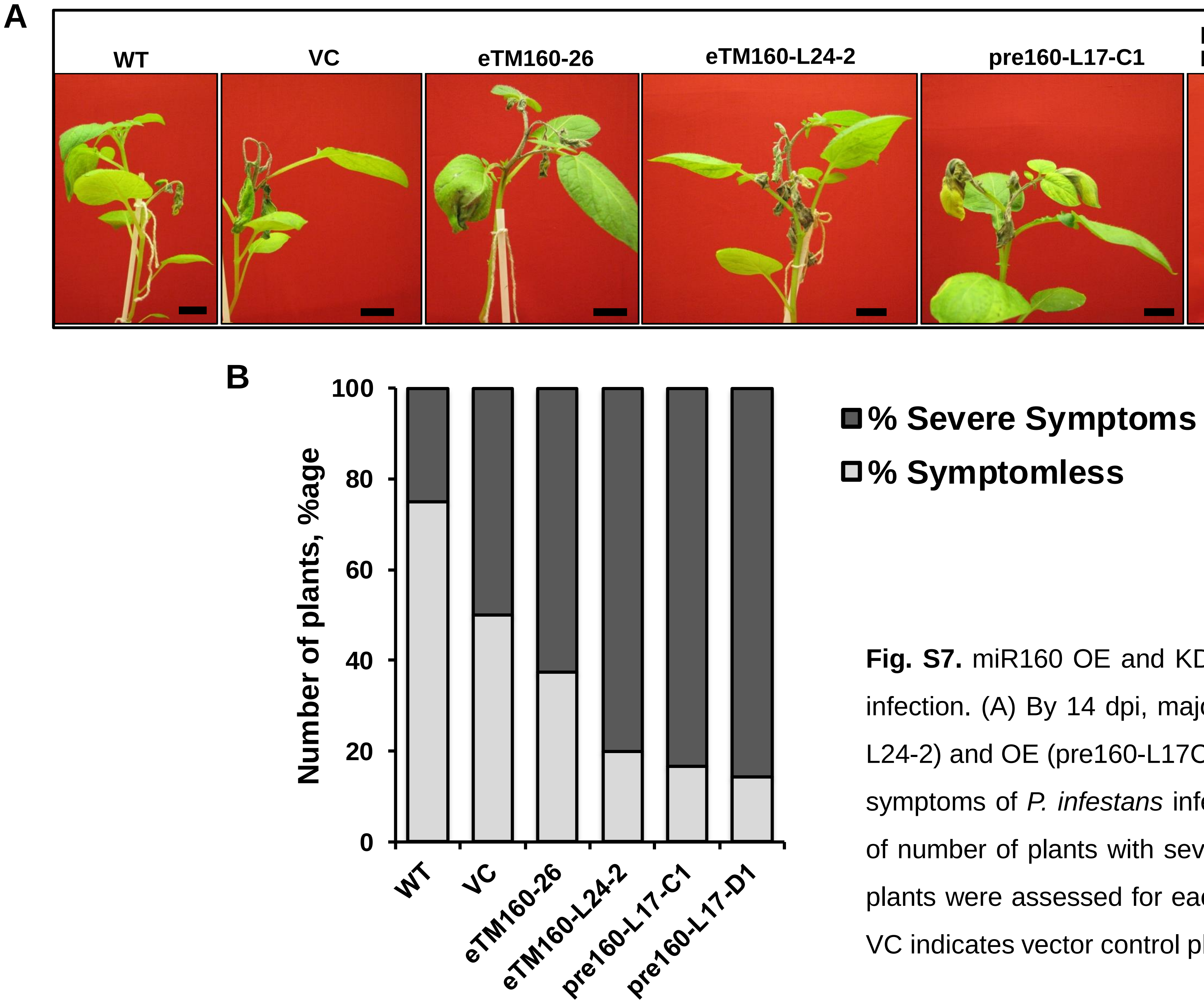

Fig. S7. miR160 OE and KD lines are highly susceptible to *P. infestans* infection. (A) By 14 dpi, majority of miR160 KD (eTM160-26 & eTM160-L24-2) and OE (pre160-L17C1 and pre160-L17D1) plants showed severe symptoms of *P. infestans* infection. Scale bar of 2 cm. (B) Quantification of number of plants with severe disease symptoms. Four to ten infected plants were assessed for each plant type. WT indicates wild-type plants, VC indicates vector control plants.

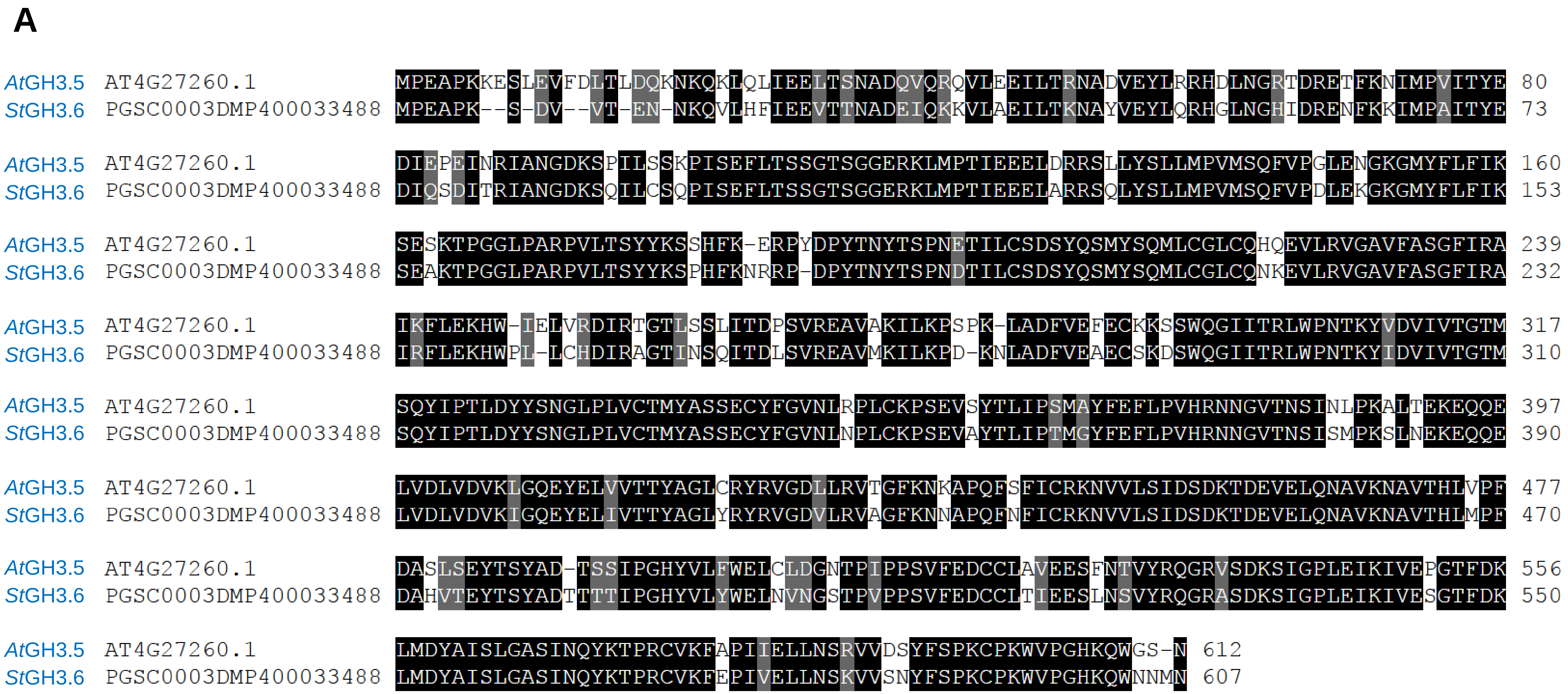

B

Percent Similarity	Percent Coverage	E value
77.6	99	$1.4e^{-206}$

Fig. S8. Sequence similarity between *At*GH3.5 and *St*GH3.6 protein sequences. (A) Pairwise sequence alignment of *At*GH3.5 and *St*GH3.6. The dark background indicates amino acid conservation between the two sequences. (B) The percent similarity between *At*GH3.5 and *St*GH3.6 protein sequences as obtained from BLAST alignment in PGSC database.

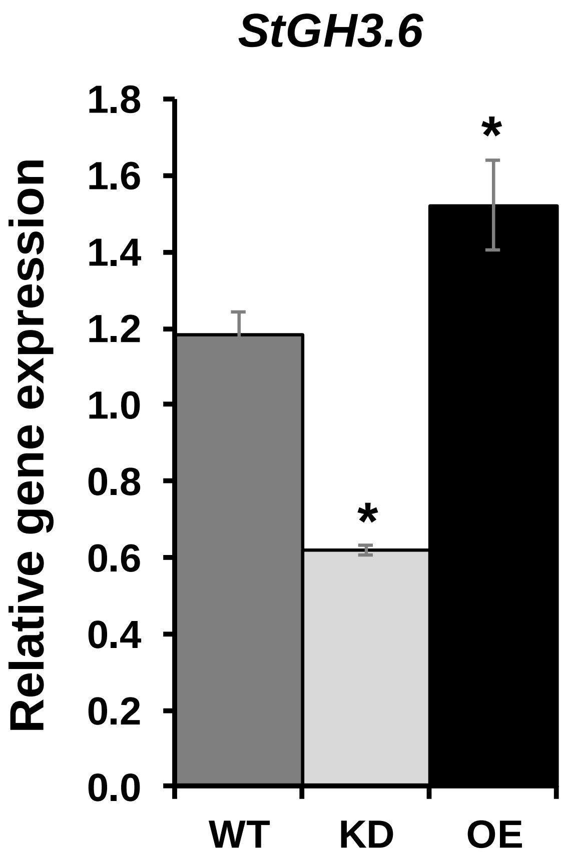

Fig. S9. Expression of *StGH3.6* in miR160 transgenic lines. Levels of *StGH3.6* in miR160 knockdown line (KD, eTM160-26) and overexpression line (OE, pre160-17-LC1) compared to wild-type (WT) plants under non-infective conditions. Data represents mean ± SE (standard error) of three biological replicates having three technical replicates each. Analysis was carried out by comparing KD and OE to WT samples. Asterisks indicate significant difference between the two bars being compared as per Student's t-test ($p<0.05$).

A

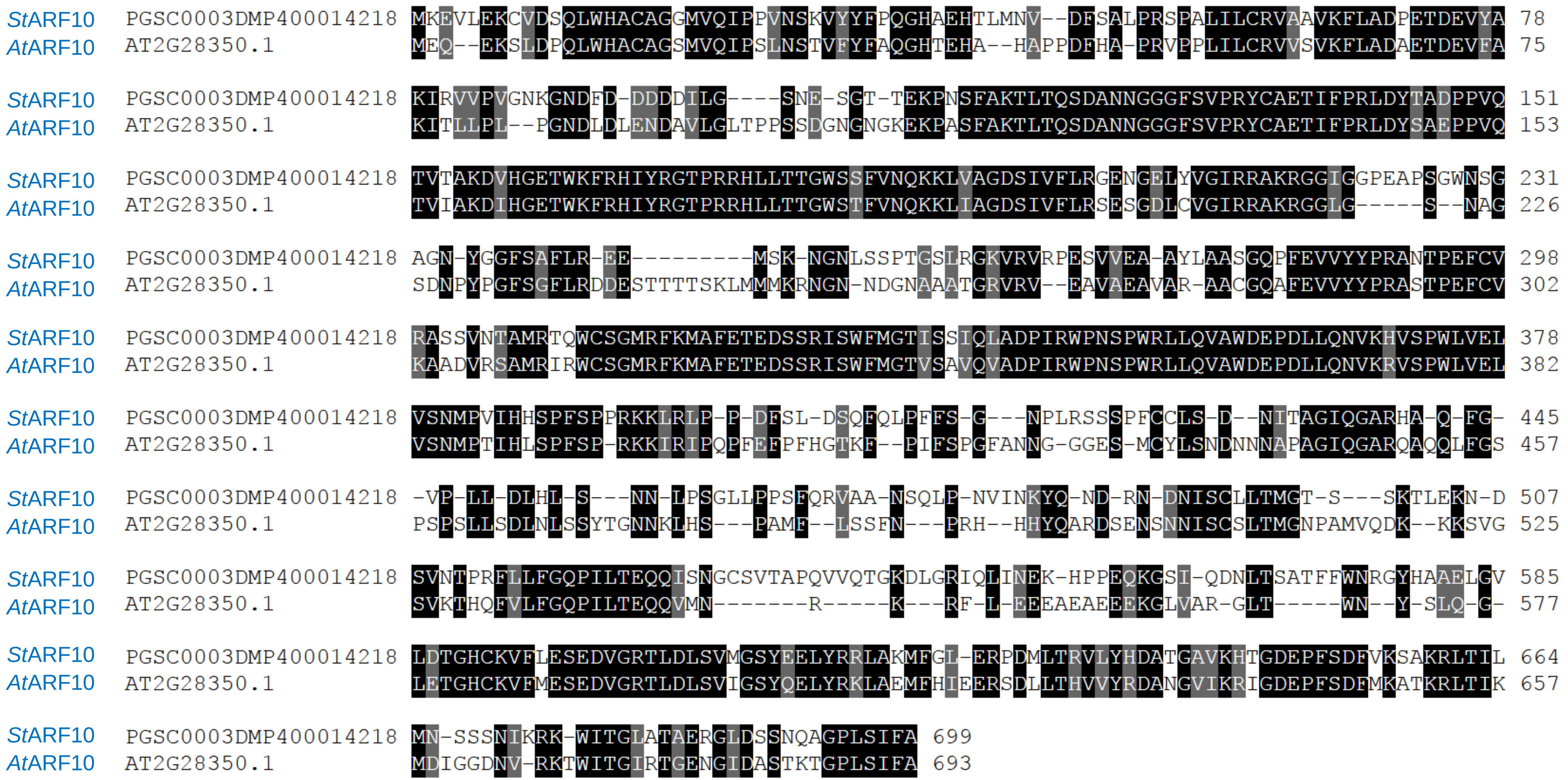

B

Percent Similarity	Percent Coverage	E value
70.3	99	$9.6e^{-205}$

Fig. S10. Sequence similarity between *St*ARF10 and *At*ARF10 protein sequences**.** (A) Pairwise sequence alignment of *St*ARF10 and *At*ARF10 . The dark background indicates amino acid conservation between the two sequences. (B) The percent similarity between *St*ARF10 and *At*ARF10 protein sequences as obtained from BLAST alignment in PGSC database.

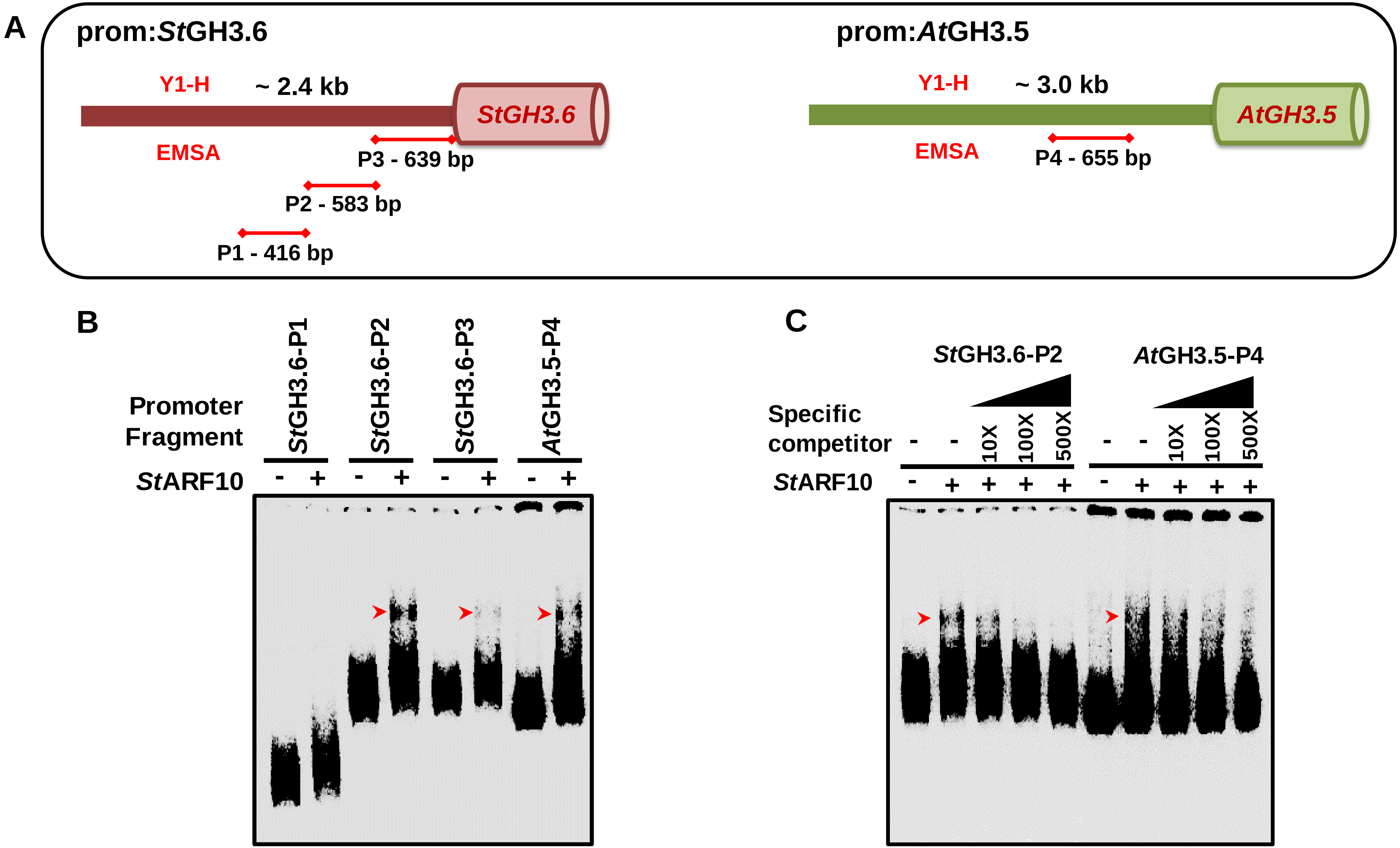

A
prom:StGH3.6
Y1-H
~ 2.4 kb
StGH3.6
EMSA
P3 - 639 bp
P2 - 583 bp
P1 - 416 bp
prom:AtGH3.5
Y1-H
~ 3.0 kb
AtGH3.5
EMSA
P4 - 655 bp
B
Promoter Fragment
StGH3.6-P1
StGH3.6-P2
StGH3.6-P3
AtGH3.5-P4
StARF10
- + - + - + - +
C
StGH3.6-P2
AtGH3.5-P4
Specific competitor
- - 10X 100X 500X - - 10X 100X 500X
StARF10
- + + + + - + + + +

Fig. S11. Electrophoretic mobility shift assay (EMSA) to understand the interaction of StARF10 protein with promoter fragments of *StGH3.6* and *AtGH3.5*. (A) Promoter fragments used for EMSA and their comparison with the promoter used for Y1-H analysis: (1) From prom:*StGH3.6,* promoter fragments P1 (416 bp, -1191 to -1607), P2 (583 bp, -620 to -1204) and P3 (639 bp, -1 to -639) were used; (2) From prom:*AtGH3.5*, fragment P4 (655 bp, -624 to -1278) was used. (B) EMSA binding assays showing the interaction (red arrows pointing the shifted bands) of recombinant *St*ARF10 with P2 and P3 fragments of prom:*St*GH3.6 and P4 fragment of prom:*At*GH3.5. (C) Cold competition assay performed with increasing molar concentrations of unlabelled P2 and P4 further confirmed the interaction.

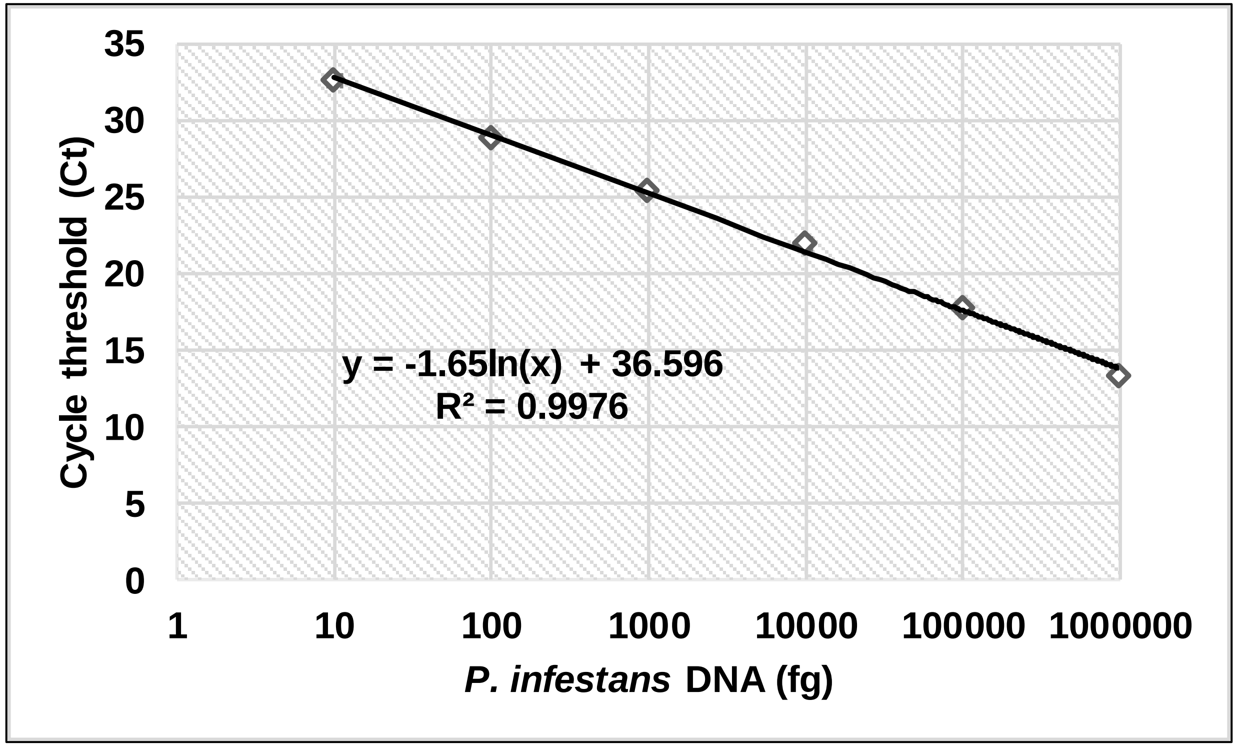

Fig. S12. Standard graph for absolute quantification of *P. infestans* biomass. Standard curve was prepared with different concentrations of *P. infestans* genomic DNA for amplification of the O8 repetitive sequence with O8-3 and O8-4 primers. fg indicates femtograms.

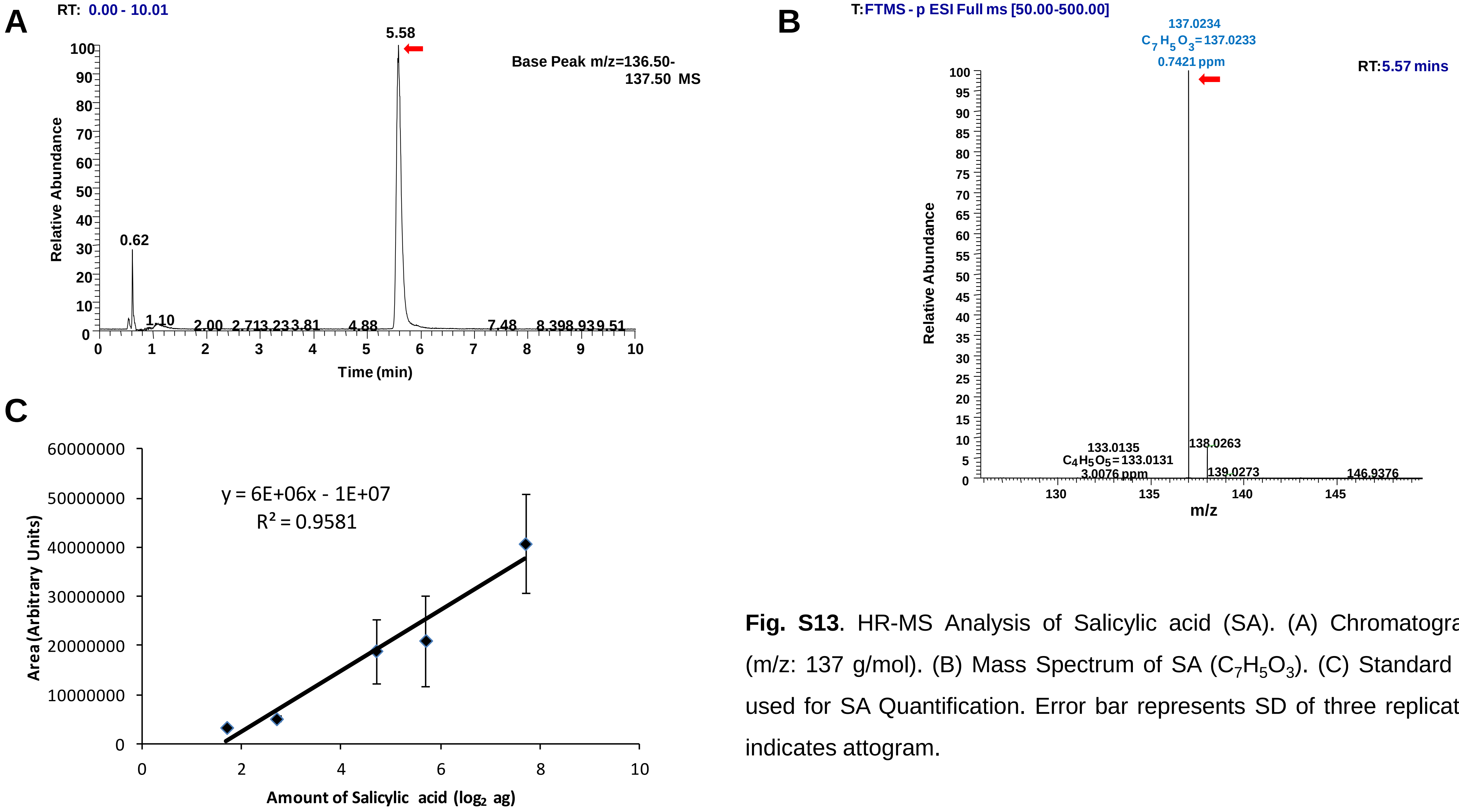

Fig. S13. HR-MS Analysis of Salicylic acid (SA). (A) Chromatogram SA (m/z: 137 g/mol). (B) Mass Spectrum of SA ($C_7H_5O_3$). (C) Standard Graph used for SA Quantification. Error bar represents SD of three replicates. ag indicates attogram.

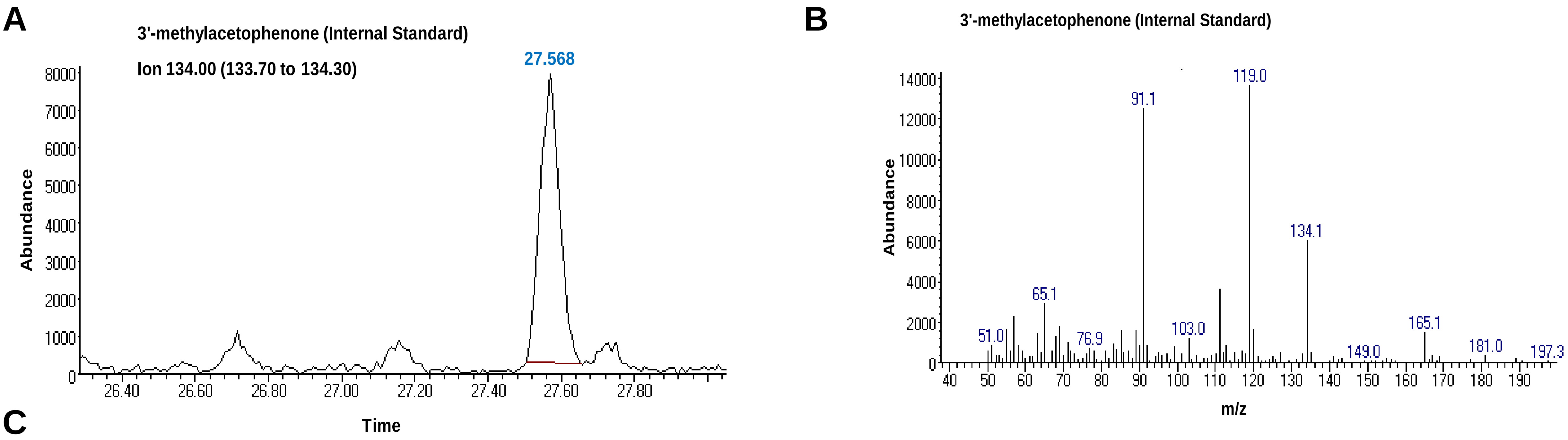

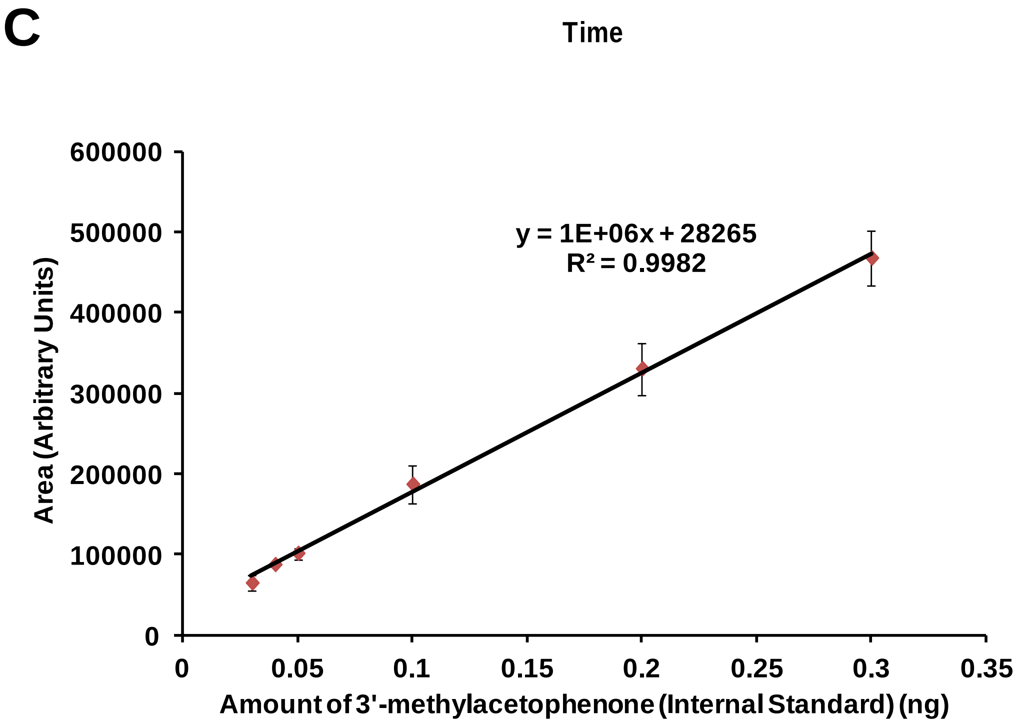

Fig. S14. GC-MS Analysis of Internal Standard, 3'-methylacetophenone**.** (A) Chromatogram of 3'-methylacetophenone (m/z: 134 g/mol). (B) Mass Spectrum of 3'-methylacetophenone ($C_9H_{10}O$). (C) Standard Graph of 3'-methylacetophenone. Error bar represents SD of three replicates. ng indicates nanogram.

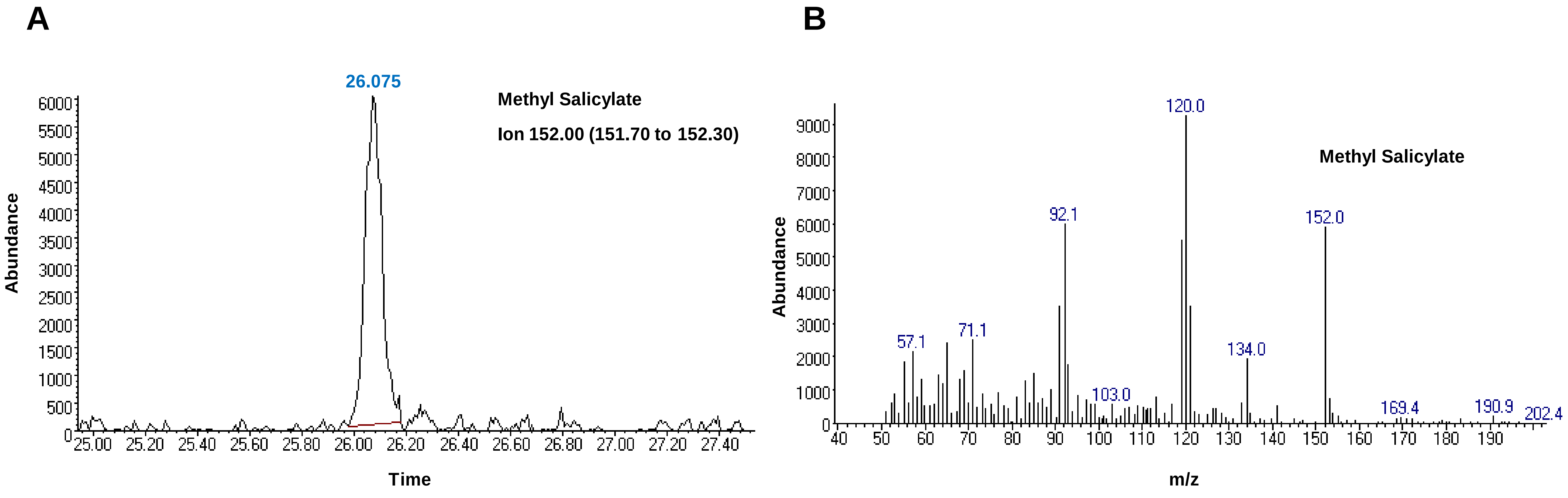

Fig. S15. GC-MS Analysis of Methyl Salicylate (MeSA). (A) Chromatogram of MeSA (m/z: 152 g/mol). (B) Mass Spectrum of MeSA ($C_8H_8O_3$).
