# Supplementary material for: MiRNA160 is associated with local defense and systemic acquired resistance against Phytophthora infestans infection in potato
Source: J Exp Bot. 2018 Jan 30;69(8):2023–36. doi: 10.1093/jxb/ery025 (PMC6018911; doi:10.1093/jxb/ery025)
Supplement: Supplementary Tables S1-S2 [file ery025_suppl_supplementary_tables_s1-s2.pdf]

Table S1. Target prediction analysis of miR160 from potato

S. No.	Target Name in our study	PGSC or NCBI Accession No.	Name of the Target in PGSC/NCBI	Accession of Arabidopsis Homolog from TAIR	Expectation value (E-value)		
					psRNA Target	Target align	TAPIR
1	Auxin response factor 10, *StARF10*	PGSC0003DMT400020874	Auxin Response Factor ARF10	AT2G28350.1	0	0	0
2	Auxin response factor 16, *StARF16*	PGSC0003DMT400062489	Auxin Response Factor ARF16	AT4G30080.1	0	0	0
3	*StARF16 - 2*	PGSC0003DMT400045323	auxin response factor ARF16	AT4G30080.1	0.5	0.5	0.5
4	*StARF10 - 2*	PGSC0003DMT400055614	Auxin response factor 10	No match	1.5	1	2
5	*StARF17*	XM_006364896	auxin response factor 17	AT1G77850.1	0.5	---	---
6	*StMAPK9*	PGSC0003DMT400015264	MAPK9	AT3G18040.1	3	---	---

PGSC: Potato Genome Sequencing Consortium
NCBI: National Center for Biotechnology Information
TAIR: The Arabidopsis Information Resource

Table S2. Details of the primers used in this study

Primer Name	Sequence 5' - 3'
**Mature miR detection and qPCR**	
**miR160-STP**	GTCGTATCCAGTGCAGGGTCCGAGGTATTCGCACTGGATACGACGGCATA
**miR166-STP**	GTCGTATCCAGTGCAGGGTCCGAGGTATTCGCACTGGATACGACGGGAAT
**miR159-STP**	GTCGTATCCAGTGCAGGGTCCGAGGTATTCGCACTGGATACGACTAGAGC
**miR169-STP**	GTCGTATCCAGTGCAGGGTCCGAGGTATTCGCACTGGATACGACTAGGCA
**miR172-STP**	GTCGTATCCAGTGCAGGGTCCGAGGTATTCGCACTGGATACGACTGCAGC
**miR396-STP**	GTCGTATCCAGTGCAGGGTCCGAGGTATTCGCACTGGATACGACAGTTCA
**miR156-STP**	GTCGTATCCAGTGCAGGGTCCGAGGTATTCGCACTGGATACGACGTGCTC
**miR171-STP**	GTCGTATCCAGTGCAGGGTCCGAGGTATTCGCACTGGATACGACGATATT
**miR414-STP**	GTCGTATCCAGTGCAGGGTCCGAGGTATTCGCACTGGATACGACTGACGA
**miR1533-STP**	GTCGTATCCAGTGCAGGGTCCGAGGTATTCGCACTGGATACGACTCATAA
**miR160-FP**	TGGAGTTTGCCTGGCTCCCTG
**miR166-FP**	TGGAGGTTCGGACCAGGCTTC
**miR159-FP**	CGGCGGTTTGGATTGAAGGGA
**miR169-FP**	CGGCGGTTAGCCAAGGATGACT
**miR172-FP**	CGGCGGCAGAATCTTGATGAT
**miR396-FP**	CGGCGGTTTCCACAGCTTTCT
**miR156-FP**	GCGGCGGTGACAGAAGAGAGT
**miR171-FP**	TGTGGATTGATTGAGCCGCGCC
**miR414-FP**	CGGCGGCTCATCTAGATCATCA
**miR1533-FP**	GCGGCGGCATAAAAAAAATAAT
**Univ-miR-RP**	AGTGCAGGGTCCGAGGT

**Northern Blot Analysis**	
**miR160_RC**	GGCATACAGGGAGCCAGGCA
**U6_RC**	AGGGGCCATGCTAATCTTCTC
**miRNA Precursor Detection, miR160 Overexpression, Clone Confirmation, qRT-PCR**	
***St*-pre160-FP**	GAGATCTAGACACGTCGTGTACACGTATA
***St*-pre160-RP**	GAGAGAGCTCCAACATCATATACACGATATCGG
**miR160 Knockdown, Clone Confirmation, qRT-PCR**	
**eTM160-FP**	TCTTCAGAGATGGCCTGAC
**eTM160-RP**	AATCGTAATCCTAATCAGTGTT
**MIM-FP**	GAGACCCGGGAAAACACCACAAAAACAAAAGA
**MIM-RP**	GAGAGAGCTCAAGAGGAATTCACTATAAAGAG
**NosT-RP**	GCAACAGGATTCAATCTTAAG
**miRNA Target Detection, Cleavage Site Mapping and qRT-PCR**	
***St*ARF10-FP**	GTCCAGCAGTCCTTTCTGTTGTTT
***St*ARF10-RP1**	GGCTGACCGAAGAGTAAGAACC
***St*ARF10-RP2**	GCTGCAACACGCTGGAAACTT
***St*ARF16-FP**	GGCAACCCCCTCAGGTCTAG
***St*ARF16-RP1**	GCATCAACTTGTTGGGAAGCGG
***St*ARF16-RP2**	TGCAACTTTTCGCTACGGTGGA
**qRT-PCR of Auxin Pathway Genes**	
***St*LAX4_FP**	TTAACTCTGCTGTTGGGGCTCT
***St*LAX4_RP**	CTTGGCATGAAGGATGGTGG
***St*TIR1_FP**	AACCCTGAGCTTGGCAAGC
***St*TIR1_RP**	GGCCTTGCTCCGTCAAGGTT
***St*YUCCA1_FP**	AAATTAGGTCTCCGGCGA
***St*YUCCA1_RP**	TTTCCTTCACACCTGGCAT

***St*IAA16_FP**	GAAGACAAGGATGGTGATTGGA
***St*IAA16_RP**	TCCACTGCTCTTGGTGCTA
***St*GH3.6_FP**	AAGTCCATCGGGCCATTGGA
***St*GH3.6_RP**	CCATTTTGGGCATTTGGGGCT
**qRT-PCR of Defence-related Genes**	
***St*PR1-FP**	GTACCAACCAATGTGCAAGCG
***St*PR1-RP**	TGTCCGACCCAGTTTCCAAC
***St*MES1-FP**	CATCATTGGTGAGACCAAGCTC
***St*MES1-RP**	TGGTATGCCTTTGTCCTCAGT
***St*BSMT1-FP**	GAGTGCCTGGTTCATTTTATAC
***St*BSMT1-RP**	GGACTTGTACTTGCCATGTAA
***St*NPR1-FP**	AAGAGGCTCACTAGGCTT
***St*NPR1-RP**	GCTTCATACGCAAATCATCG
**Y1-H Analysis**	
**ARF10_Y1H_FP**	AAAAAGCAGGCTTCATGAAGGAGGTTTTGGAGAAGT
**ARF10_Y1H_RP**	CAAGAAAGCTGGGTTCTATGCAAAGATGCTAAGAGG
**ATTB1_Y1H_FP**	GGGGACAAGTTTGTACAAAAAAGCAGGCT
**ATTB2_Y1H_RP**	GGGGACCACTTTGTACAAGAAAGCTGGGT
**Prom-*St*GH3.6_Y1H_F**	TATAGAAAAGTTGTCAATGGTAGTACCCCACG
**Prom-*St*GH3.6_Y1H_R**	TTTGTACAAACTTGCGGTTTCTTTTAATTAACAAAAGTGAACA
**Prom-*At*GH3.5_Y1H_F**	TATAGAAAAGTTGTCTTTTAAATTAACTAAGTTCGATAAACTGTG
**Prom-*At*GH3.5_Y1H_R**	TTTGTACAAACTTGCGGTTTAAGAGAAAGAGAGAAGTC
**p53_Y1H_F**	TATAGAAAAGTTGTCTACCAGGCATGCCTAGCA
**p53_Y1H_R**	TTTGTACAAACTTGCATACAGAGCACATGCCTC
**ATTB4_FP**	GGGGACAACTTTGTATAGAAAAGTTGTC
**ATTB1_RP**	GGGGACTGCTTTTTTGTACAAACTTGC

**EMSA**	
***St*ARF10_F_FP**	GGATCCATGAAGGAGGTTTTGGAGAAGTGT
***St*ARF10_F_RP**	AAGCTTTGCAAAGATGCTAAGAGGTCCA
***St*GH3.6-P1_FP**	GGATCCGCAGGTAACGTGTCTATTT
***St*GH3.6-P1_RP**	GAATTCGCGACTTAGAGTACGTATT
***St*GH3.6-P2_FP**	GGATCCAATACGTACTCTAAGTCGC
***St*GH3.6-P2_RP**	GAATTCTAGTTGGTGAGTTAGATCG
***St*GH3.6-P3_FP**	GGATCCCGATCTAACTCACCAACTA
***St*GH3.6-P3_RP**	GAATTCTGTGAAGAAAAAGAGAGAGTTTG
***At*GH3.5-P4_FP**	GGATCCCTATCAAGTTTGGAGTCCA
***At*GH3.5-P4_RP**	CCCGGGATTGCAGTGTAGTTGGTAC
**Pathogen Detection**	
**PINF**	CTCGCTACAATAGGAGGGTC
**ITS5**	GGAAGTAAAAGTCGTAACAAGG
**Rs-BP4R**	GACGACATCATTTCCACCGGGCG
**Rs-BP4L**	GGGTGAGATCGATTGTCTCCTTG
**Reference Gene for qRT-PCR**	
**GAPDH-FP**	GAAGGACTGGAGAGGTGGA
**GAPDH-RP**	GACAACAGAAACATCAGCAGT
